# Supplementary material for: Validation of visual estimation of neonatal jaundice in low-income and middle-income countries: a multicentre observational cohort study
Source: BMJ Open. 2021 Dec 31;11(12):e048145. doi: 10.1136/bmjopen-2020-048145 (PMC8720979; doi:10.1136/bmjopen-2020-048145)
Supplement: Supplementary data [file bmjopen-2020-048145supp001.pdf]

Table S1. Primary diagnoses of infants 1-20 days of age who presented to Young Infant Clinical Signs Study Group sites for evaluation

Primary diagnosis	N	%	Cumulative %
Jaundice	756	28.6	28.6
URTI / mild ARI	233	8.8	37.4
Skin infection	195	7.4	44.8
Eye infection	189	7.2	52.0
Sepsis	166	6.3	58.2
Feeding problem	94	3.6	61.8
Gastrocolic reflex	83	3.1	65.0
Birth asphyxia	65	2.5	67.4
Congenital malformation	61	2.3	69.7
Skin condition (non-infectious)	59	2.2	72.0
Birth injury	58	2.20	74.2
Colic	48	1.8	76.0
Diarrhea	45	1.70	77.7
Prematurity	41	1.6	79.2
Umbilical infection	41	1.6	80.8
Pneumonia / ALRI	37	1.4	82.2
Oral thrush	29	1.1	83.3
Cephalohematoma	25	1.0	84.2
Nasolacrimal duct block	20	0.8	85.0
High environmental temperature	17	0.6	85.6
Regurgitation	16	0.6	86.2
Umbilical problem	16	0.6	86.8
Low birth weight	14	0.5	87.4
Seizures	12	0.4	87.8
Breast abscess	12	0.4	88.3
Meningitis	9	0.3	88.6
Constipation	9	0.3	89.0
Hypoglycaemia	5	0.2	89.1

Laryngomalacia	5	0.2	89.3
Transient tachypnoea	3	0.1	89.4
Meconium aspiration syndrome	3	0.1	89.6
Hypothermia	3	0.1	89.7
Dysentery (bloody stools)	3	0.1	89.8
Intestinal obstruction	3	0.1	89.9
None	150	5.7	96.1
Other (≤ 2 cases)	117	4.4	---
Total	2,642	100.0	

*Note that percentages are rounded off to the nearest 0.1; they add to 100% over the entire sample

Abbreviations: ALRI, acute lower respiratory infection; ARI, acute respiratory infection; URTI, upper respiratory tract infection
