# Supplementary material for: Validation of visual estimation of neonatal jaundice in low-income and middle-income countries: a multicentre observational cohort study
Source: BMJ Open. 2021 Dec 31;11(12):e048145. doi: 10.1136/bmjopen-2020-048145 (PMC8720979; doi:10.1136/bmjopen-2020-048145)
Supplement: Author's manuscript [file bmjopen-2020-048145.draft_revisions.pdf]

BMJ Open

BMJ Open is committed to open peer review. As part of this commitment we make the peer review history of every article we publish publicly available.

When an article is published we post the peer reviewers' comments and the authors' responses online. We also post the versions of the paper that were used during peer review. These are the versions that the peer review comments apply to.

The versions of the paper that follow are the versions that were submitted during the peer review process. They are not the versions of record or the final published versions. They should not be cited or distributed as the published version of this manuscript.

BMJ Open is an open access journal and the full, final, typeset and author-corrected version of record of the manuscript is available on our site with no access controls, subscription charges or pay-per-view fees (http://bmjopen.bmj.com).

If you have any questions on BMJ Open's open peer review process please email info.bmjopen@bmj.com

BMJ Open

Validation of clinical assessment of neonatal jaundice in low-and middle-income countries: a multicentre observational cohort study

Journal:	*BMJ Open*
Manuscript ID	bmjopen-2020-048145
Article Type:	Original research
Date Submitted by the Author:	17-Dec-2020
Complete List of Authors:	Darmstadt, Gary; Stanford University School of Medicine, Pediatrics<br>Hamer, D.; Boston University School of Public Health, Department of Global Health<br>Carlin, John; Murdoch Childrens Research Institute, Clinical Epidemiology and Biostatistics Unit<br>Jeena, Prakash; University of KwaZulu-Natal Nelson R Mandela School of Medicine, Department of Paediatrics & Child Health<br>Mazzi, Eduardo; Hospital del Nino Dr Ovidio Aliaga Uria<br>Narang, Anil; Post Graduate Institute of Medical Education and Research<br>Deorari, A; All India Institute of Medical Sciences, Pediatrics<br>Addo-Yobo, Emmanuel; Kwame Nkrumah University of Science and Technology, School of Medical Sciences<br>Chowdhury, MAK Azad; Dhaka Shishu Hospital, Neonatology<br>Kumar, Praveen; Post Graduate Institute of Medical Education and Research<br>Abu-Sarkodie, Yaw; Kwame Nkrumah University of Science and Technology<br>Yeboah-Antw, K.; Kwame Nkrumah University of Science and Technology<br>Ray, Pallab; Post Graduate Institute of Medical Education and Research<br>Bartos, Andres; Hospital Materno-Infantil, Pediatrics<br>Saha, Samir K.; Child Health Research Foundation<br>Foote, Eric; Stanford University School of Medicine, Pediatrics<br>Bahl, Rajiv; World Health Organization, Department of Maternal, Newborn, Child and Adolescent Health<br>Weber, Martin; World Health Organization Regional Office for Europe
Keywords:	NEONATOLOGY, Community child health < PAEDIATRICS, PUBLIC HEALTH

SCHOLARONE™
Manuscripts

I, the Submitting Author has the right to grant and does grant on behalf of all authors of the Work (as defined in the below author licence), an exclusive licence and/or a non-exclusive licence for contributions from authors who are: i) UK Crown employees; ii) where BMJ has agreed a CC-BY licence shall apply, and/or iii) in accordance with the terms applicable for US Federal Government officers or employees acting as part of their official duties; on a worldwide, perpetual, irrevocable, royalty-free basis to BMJ Publishing Group Ltd ("BMJ") its licensees and where the relevant Journal is co-owned by BMJ to the co-owners of the Journal, to publish the Work in this journal and any other BMJ products and to exploit all rights, as set out in our licence.

The Submitting Author accepts and understands that any supply made under these terms is made by BMJ to the Submitting Author unless you are acting as an employee on behalf of your employer or a postgraduate student of an affiliated institution which is paying any applicable article publishing charge ("APC") for Open Access articles. Where the Submitting Author wishes to make the Work available on an Open Access basis (and intends to pay the relevant APC), the terms of reuse of such Open Access shall be governed by a Creative Commons licence – details of these licences and which Creative Commons licence will apply to this Work are set out in our licence referred to above.

Other than as permitted in any relevant BMJ Author's Self Archiving Policies, I confirm this Work has not been accepted for publication elsewhere, is not being considered for publication elsewhere and does not duplicate material already published. I confirm all authors consent to publication of this Work and authorise the granting of this licence.

For peer review only

Validation of clinical assessment of neonatal jaundice in low-and middle-income countries: a multicentre observational cohort study

Running title: Multicentre clinical assessment of neonatal jaundice

Gary L. Darmstadt[1a], Davidson H. Hamer[2,3], John B. Carlin[4], Prakash M. Jeena[5], Eduardo Mazzi[6], Anil Narang[7], Ashok Deorari[8], Emmanuel Addo-Yobo[9], MAK Azad Chowdury[10], Praveen Kumar, DM[7], Yaw Adu-Sarkodie[9], Kojo Yeboah-Antwi[9], Pallab Ray[7], Andrés Bartos[11], Samir K. Saha[12], Eric M. Foote[1], Rajiv Bahl,[13] Martin W. Weber[14], for the Young Infants Clinical Signs Study (YICSS) Group

1. Department of Pediatrics, Stanford University School of Medicine, Stanford, CA, USA;
2. Department of Global Health, Boston University School of Public Health, Boston, MA, USA
3. Section of Infectious Diseases, Department of Medicine, Boston University School of Medicine, Boston, MA, USA
4. Clinical Epidemiology and Biostatistics Unit, Murdoch Children's Research Institute and The University of Melbourne, Melbourne, Australia
5. Department of Paediatrics & Child Health, Nelson R. Mandela School of Medicine, University of KwaZulu-Natal, Durban, South Africa
6. Department of Pediatrics, Hospital del Niño "Dr. Ovidio Aliaga Uría", La Paz, Bolivia
7. Post Graduate Institute of Medical Education and Research, Chandigarh, India
8. Department of Pediatrics, Division of Neonatology, All India Institute for Medical Sciences, Delhi, India

For peer review only - http://bmjopen.bmj.com/site/about/guidelines.xhtml

9. School of Medical Sciences, Kwame Nkrumah University of Science & Technology, Kumasi, Ghana

10. Department of Neonatology, Dhaka Shishu Hospital, Dhaka, Bangladesh

11. Department of Pediatrics, Hospital Materno Infantil, La Paz, Bolivia

12. Child Health Research Foundation, Dhaka, Bangladesh

13. Newborn Health Unit, World Health Organization, Geneva, Switzerland

14. World Health Organization Regional Office for Europe, Copenhagen, Denmark

[a] Previous affiliation: Department of International Health, Johns Hopkins School of Public Health, Baltimore, MD, USA

Correspondence: Gary L. Darmstadt, MD, MS, Department of Pediatrics, Stanford University School of Medicine, 1701 Page Mill Road, Palo Alto, CA 94304, USA, gdarmsta@stanford.edu, tel: 650-724-6014

Key words: Jaundice, algorithm, hyperbilirubinemia, neonatal morbidity, primary care

Words: Abstract, 300; text, 3618

Tables: 5; **Figures:** 1

For peer review only

Abstract

Objective: Determine the sensitivity and specificity of neonatal jaundice algorithms as assessed by primary healthcare workers (PHW) and physicians as predictors of hyperbilirubinemia.

Design: Multiccenter observational cohort study.

Setting: Health facilities in Chandigarh and Delhi, India; Dhaka, Bangladesh; Durban, South Africa; Kumasi, Ghana; La Paz, Bolivia.

Participants: Neonates aged 1-20 days (n=2642) who presented to hospitals for any type of acute illness. Infants referred or needing immediate cardiopulmonary resuscitation were excluded.

Outcome measures: Infants were evaluated for distribution (head, trunk, distal extremities) and degree (mild, moderate, severe) of jaundice by PHWs and physicians. Serum bilirubin level was determined for infants with jaundice, and analyses of sensitivity and specificity of clinical classifications of jaundice utilised bilirubin thresholds of >260 μmol/L (need for phototherapy) and >340 μmol/L (need for plasma exchange transfusion in at-risk and preterm babies).

Results: 1241 (47.0%) neonates had jaundice. High sensitivity for detecting neonates with serum bilirubin >340 μmol/L was found for the algorithm "any jaundice of the distal extremities (palms or soles) or deep jaundice of the trunk or head" for both PHWs (89-100%) and physicians (81-100%) across study sites; specificity was more variable. "Any jaundice of the distal extremities" identified by PHWs and physicians had sensitivity of 71-100% and specificity of 55-95%, excluding La Paz. For the bilirubin threshold >260 μmol/L, "any jaundice of the distal extremities or deep jaundice of the trunk or head" had the highest sensitivity across sites (PHWs: 58-93%, physicians: 55-98%).

For peer review only - http://bmjopen.bmj.com/site/about/guidelines.xhtml

Conclusions: In settings where serum bilirubin cannot be measured, neonates with any jaundice on the distal extremities should be referred to a hospital for evaluation and management. Where delays in serum bilirubin measurement and appropriate treatment are anticipated following referral, the higher-sensitivity algorithm, any jaundice on the distal extremities or deep jaundice of the trunk or head, may be preferred.

Article Summary

Strengths and limitations of this study'

- This study was designed to address the evidence gap from low- to middle-income countries on the reliability of clinical signs to screen newborn infants for jaundice in the first three weeks after delivery to determine the need for facility-based care, and is the largest evaluation reported to date, to our knowledge, to compare clinical assessments for jaundice in newborn infants by primary health care workers and physicians.
- The findings have the potential to be generalised to large populations in low- and middle-income countries and to influence World Health Organisation guidelines.
- We did not objectively assess the depth of jaundice, and 22% of infants with some degree of jaundice did not have laboratory work done.
- Asymptomatic infants with jaundice may not have been brought for assessment, and thus, the sensitivity and specificity of clinical assessments may vary at population level.
- **T**his study did not include sufficient numbers of preterm infants to allow for independent assessment of the utility of jaundice detection in this subgroup of infants who are especially vulnerable to morbidity and mortality from hyperbilirubinemia

For peer review only

Introduction

Jaundice is among the most common neonatal problems, particularly in the first week.[1] Delayed detection and therapy place newborn infants, especially those born preterm, at risk for bilirubin encephalopathy and kernicterus, leading to neurodevelopmental impairment, disability and potentially death.[2-5] Globally in 2010 an estimated 481,000 newborn infants were at risk of extreme hyperbilirubinemia, and of these, 24% were at risk of neonatal death, 13% for kernicterus and 11% for stillbirth.[6]

Prevention of morbidity and mortality due to hyperbilirubinemia in low- and middle-income countries could markedly improve if primary healthcare health workers (PHWs) were able to accurately identify and appropriately refer newborn infants with clinically significant jaundice. The Integrated Management of Childhood Illness (IMCI) strategy to identify young infants with severe illness needing urgent referral-level care was revised in 2008 based on the results of the Young Infant Clinical Signs Study (YICSS),[1] and was further validated in a community-based setting in Bangladesh.[7] In 2014, IMCI included jaundice of palms and soles as a need for urgent referral.[8] Approximately three-fourths of countries have included jaundice of palms and soles in their use of IMCI, based largely on expert opinion.[9] Jaundice on the day of childbirth is always a pathological sign that requires referral to hospital for evaluation and management. Also, jaundice persisting or first presenting more than three weeks after delivery is generally pathological, and these infants also need referral for evaluation. A major gap in IMCI guidelines is the need to validate clinical signs of jaundice in newborn infants aged 1-20 days which signal urgent referral for hospital-level care.

For peer review only

Several studies have examined the correlation between identification of jaundice in newborn infants by experienced physicians and serum bilirubin levels.[10-16] The extent of peripheral spread of jaundice has been advocated as a useful clinical sign for identifying infants with high levels of serum bilirubin needing intervention.[10-12,14-17] However, others have questioned the ability of healthcare providers, including physicians and nurses, to diagnose clinical jaundice[18,19] and data are lacking on the ability of PHWs in LMIC settings to identify newborn infants with clinically significant jaundice requiring referral for care. Point-of-care technologies increasingly show promise as an alternative approach for identifying hyperbilirubinemia, but are not widely available.[20-22]

In this multicentre study nested in the YICSS,[1] we determined the sensitivity and specificity of a range of definitions of clinical jaundice, according to distribution and severity of skin staining as assessed by PHWs and by physicians, and the agreement between their clinical assessments, for predicting the presence of serum hyperbilirubinemia in neonates in the first three weeks.

Methods

Study locations and patient selection

Each of the six study locations over three continents (Table 1) collected data for a consecutive 12-month period during 2003 to 2005, as described previously.[23-26] Young infants brought to the hospital for any type of acute illness were included in the main study if they were <60 days of age and provided informed consent, as described previously, including sample size determination.[1] Recruitment was stratified into two age categories – 0-6 and 7-59 days – to ensure adequate sampling of neonates in the first week, when data are particularly lacking.[27]

For peer review only - http://bmjopen.bmj.com/site/about/guidelines.xhtml

Infants presenting for care could only be enrolled once and, to ensure follow-up, had to reside in the defined study area. We excluded young infants who: 1) needed immediate cardiopulmonary resuscitation upon presentation to the hospital, 2) had an obvious lethal congenital abnormality, 3) had been hospitalised in the previous two weeks (except for delivery), or 4) were referred from another health facility.

Table 1. Methods of bilirubin detection and quality assurance by study location, Young Infants Clinical Signs Study Group.

Study location	Method for bilirubin measurement	Quality control measures
1) Dhaka Shishu Hospital, Dhaka, Bangladesh	Dimethylsulfoxide (Chronolab AG, Zug, Switzerland) colorimetric method using a Humalyzer - 2000 (Human Gesellschatt for Biochemica and Diagnostic mbH, Germany)	Quality control was done daily using calibration and quality control serum from the manufacturer with a range of 3.3-5.6 mg/dL
2) All India Institute of Medical Sciences and Safdarjung Hospital, Delhi, India;<br>3) Postgraduate Institute for Medical Education and Research and General Hospital, Sector 16, Chandigarh, India	Spectrophotometer (BIL-100, Cosmo Medical Co., Ltd., Seoul, Korea) with built-in auto-calibration	Bilirubin standards were used periodically (every 4-5 months) to cross-check the calibration
4) King Edward VIII Hospital, Durban, South Africa	Calorimetric method using a Roche Modular P-800 spectrophotometer (Roche Modular 800 (Roche Diagnostics, Basel, Switzerland)	The machine was manually calibrated whenever a new kit was inserted or when the machine sent out an automated request for calibration, approximately once weekly
5) Komfo Anokye Teaching Hospital, Kumasi, Ghana	Automated colorimetric method using a spectrophotometric autoanalyser (Atac-8000, Elan Diagnostics, Smithfield, RI, USA)	The machine was calibrated daily with the manufacturer's controls
6) Hospital del Niño and Hospital Materno-Infantil, La Paz, Bolivia	Diazo method (Synchron CX5 Beckman Coulter, Inc, Fullerton, CA, USA)	Quality control was run once daily with a known sample provided by the manufacturer

For peer review only

Clinical assessment of jaundice

Enrolled subjects were referred to a trained PHW (i.e. a frontline professional health worker, typically a nurse) for initial evaluation. Study coordinators worked with site principle investigators to standardise their clinical assessments within and across sites. Site principal investigators in turn trained, supervised, and attempted to standardise PHWs in their assessments of jaundice. PHWs assessed infants unclothed under well-lit conditions for presence of jaundice in three body regions: 1) head, including face, gums or sclera, 2) trunk, including chest or abdomen, and 3) distal extremities, including palms or soles. If jaundice was present at any of the sites within a body region, the degree of jaundice was scored as mild or deep, taking the deepest level of staining for the sites within the body region. At Durban, the degree of jaundice was scored as mild, moderate or severe, but for analysis, moderate and severe degrees of jaundice were combined to form the "deep" category. At Kumasi, a change in protocol midway through the study meant that limited data were available from the face and chest/abdomen in comparable form to the other study sites. Data from the La Paz study location were restricted to the first half of data collection, since the distribution of serum bilirubin levels in the second half of the study suggested the introduction of a systematic error despite quality control measures.

After the initial PHW clinical assessment, the infant was referred to a study physician for immediate evaluation and management. The physician took a complete history and performed a physical examination, blinded to the PHW's findings and to laboratory data. Signs of jaundice were assessed by physicians using the same proforma as the PHWs.

For peer review only

Laboratory evaluation of serum bilirubin

About 30-60 minutes after the study physician's evaluation, a blood sample was drawn from infants found to have any visible jaundice by the study physician and was tested for total serum bilirubin in the hospital laboratories (Table 1).

Patient management

Study physicians decided on the need for admission based on their clinical judgment and the serum bilirubin level. Admitted infants were managed according to the usual practices of the study hospitals.

Data management and analysis

Case record forms were checked for completion and double entered into an EpiData database (version 2.1, EpiData Association, Odense, Denmark) at each of the study locations. Data files were sent to the data coordination center in Melbourne, Australia, where further data cleaning and consistency checks were performed and the quality of data submitted from the individual study locations was monitored. All cases for which there was a physician assessment were included.

Description of jaundice by physicians

Box-and-whisker plots were used to display the association between increasing depth and peripheral extension of jaundice (from face to trunk to distal extremities), as assessed by physicians, and serum bilirubin level. Four categories of staining were created for display: A = No jaundice, including the head; B = Mild jaundice of the head or trunk, but none of the distal

For peer review only

extremities; C = Deep jaundice of the head or trunk, but none of the distal extremities (these data were not available for Kumasi); and D = Any jaundice of the distal extremities.

Sensitivity and specificity of jaundice definitions

A series of definitions was created based on the body site and depth of jaundice, including: (i) deep jaundice of the head, (ii) deep jaundice of the trunk, (iii) any jaundice of the distal extremities (palms, soles), (iv) any jaundice of the distal extremities OR deep jaundice of the trunk, and (v) any jaundice of the distal extremities OR deep jaundice of any site, including trunk or head. The sensitivity and specificity of classification according to these definitions of jaundice, by the PHWs and by the study physicians, were determined using thresholds for serum bilirubin as the gold standard measure as follows: (i) >260 μmol/L (>15 mg/dL), which approximates need for phototherapy according to WHO guidelines, and (ii) >340 μmol /L (>20 mg/dL), which approximates need for plasma exchange transfusion in at-risk and preterm babies.[28] All available data from PHW and physician assessments were used. A case was included if there was a response on at least one of the clinical signs; the signs that were not recorded were assumed absent.

Agreement between physicians and primary health care workers

Agreement between PHWs and physicians for identification of jaundice at each of the body regions and at each study location, for mild or severe degree of staining, was assessed by tabulating the frequency of positive classifications by each rater and using the kappa statistic. Patients aged 1-20 days were included regardless of whether they had any sign of jaundice on examination by p/ysicians or a serum bilirubin measure.

For peer review only - http://bmjopen.bmj.com/site/about/guidelines.xhtml

All data analysis was performed using Stata software, Release 10 (StataCorp, College Station, TX, USA).

Data sharing

The study protocol, deidentified participant data and statistical code will be made available upon publication of the manuscript.

Ethics

The study protocol was reviewed and approved by each of the sites' institutional review boards (IRB) or ethical committees. The Kumasi and Durban protocols were also reviewed by the Boston University Medical Center IRB. The Dhaka and La Paz protocols were reviewed by the Johns Hopkins University IRB and determined to be exempt from review because the protocol involved minimal risk to participants. The Delhi and Chandigarh protocols were reviewed by the WHO Secretariat Committee on Research Involving Human Subjects. Informed consent was obtained for all participants.

Funding

The United States Agency for International Development (USAID) provided funding for this study to the Applied Research on Child Health and Child and Family Applied Research projects at Boston University, Boston, by means of the USAID cooperative agreements (HRN-A-00-90010-00 and GHS-A-00-00020-00). The Bill and Melinda Gates Foundation provided funding for Saving Newborn Lives (SNL).

For peer review only - http://bmjopen.bmj.com/site/about/guidelines.xhtml

Patient and public involvement

Families of study subjects were not involved in study design, recruitment, or conduct or in the dissemination of study results.

Results

Prevalence of jaundice and hyperbilirubimia

Physicians assessed that 1337 of 5250 (25.5%) young infants 1-59 days of age had any sign of jaundice, 1241 of whom were aged 1-20 days (47% of 2642 babies in that age range, 93% of all young infants with jaundice) (Table 2). Among infants aged 1-20 days with any jaundice, 962 (78%) had a serum bilirubin measurement recorded; 278 (29%) and 95 (10%) had hyperbilirubinemia >260 µmol /L (>15 mg/dL) and >340 µmol /L (>20 mg/dL), respectively. The prevalence of hyperbilirubinemia varied substantially between study locations (>260 µmol /L: 10-58%, >340 µmol /L: 2-22%). Among 2608 infants aged 21-59 days (not included in this validation study), 96 (3.7%) had any sign of jaundice.

Table 2. Study profile: Infants aged 1-20 days assessed for jaundice and, among eligible neonates, numbers with serum bilirubin measured and with hyperbilirubinemia, by study location and overall.

	Study location						Overall
	Dhaka	Delhi	Chandigarh	Durban	Kumasi	La Paz	
Number assessed for jaundice*	448	330	700	414	457	293	2642
Any jaundice observed by study physicians	198	148	398	160	147	190	1241
% of assessed	44%	45%	57%	39%	32%	65%	47%
Serum bilirubin obtained	81	119	379	132	71	180	962
% of those with jaundice	41%	80%	95%	91%	48%	95%	78%
Serum bilirubin >260 µmol /l (15 mg/dL)	36	62	39	19	18	104	278
% of those with bilirubin measured	44%	52%	10%	14%	25%	58%	29%
Serum bilirubin >340 µmol /l (20 mg/dL)	14	26	9	2	8	36	95
% of those with bilirubin measured	17%	22%	2%	2%	11%	20%	10%

*By both primary healthcare workers and physicians

For peer review only - http://bmjopen.bmj.com/site/about/guidelines.xhtml

Prevalence of jaundice by depth of staining

Distributions of depth of staining by body region, according to physicians, were generally similar across locations except that the Indian physicians (especially in Delhi) recorded more deep jaundice among those found to have any jaundice (Table 3). In general, across study locations, there were decreasing proportions of subjects with deep jaundice when considering the head (22-81%) compared to the trunk (8-51%) and the distal extremities (palms/soles) (0-5%).

Table 3. Clinical signs of jaundice by body region and depth of staining among neonates aged 1-20 days with any jaundice observed by physicians, by study location.

	Infants (%) with jaundice					
	Study location					
Body region	Dhaka	Delhi	Chandigarh	Durban	Kumasi*	La Paz
Head:						
Face, gums or sclerae (*n*)	(81)	(119)	(379)	(132)	(*)	(180)
Mild	64%	19%	46%	78%		64%
Deep	36%	81%	54%	22%		36%
Trunk:						
Chest or abdomen (*n*)	(81)	(119)	(379)	(115)	(*)	(180)
None	26%	8%	24%	17%		12%
Mild	63%	40%	61%	68%		80%
Deep	11%	51%	15%	15%		8%
Distal extremities:						
Palms or soles (*n*)	(81)	(118)	(379)	(132)	(70)	(179)
None	64%	64%	93%	88%	66%	93%
Mild	35%	31%	6%	9%	34%	4%
Deep	1%	5%	1%	3%	0%	3%

* At this site, assessments were not graded as "Mild" or "Deep"; for Palms/soles the "Mild" category includes all cases with any jaundice

Association of clinical signs of jaundice with hyperbilirubinemia

Descriptive analysis

For peer review only - http://bmjopen.bmj.com/site/about/guidelines.xhtml

At all study locations, higher serum bilirubin levels were measured as severity and distal distribution of jaundice increased (Figure 1). However, this trend was more pronounced for some study locations (Delhi, Chandigarh) than for others (Kumasi), and the absolute levels of serum bilirubin corresponding to the different clinical jaundice categories varied between locations. At Dhaka, Durban and La Paz, there was relatively little difference in serum bilirubin levels between patients with deep jaundice of the head or of the trunk but none of the distal extremities (category C) compared to patients with jaundice extending to the distal extremities (category D).

Sensitivity and specificity

For the lower threshold of serum bilirubin (>260 μmol/L or >15 mg/dL), the highest overall sensitivity across sites was found for the definition "any jaundice of the distal extremities (palms/soles) or deep jaundice of the trunk or head" [PHWs: 58-93% (Table 4A), physicians: 55-98% (Table 4B)]. Sensitivity for the algorithm "any jaundice of the distal extremities" was lower for PHWs (42-88%, Table 4A), and especially for physicians (11-66%, Table 4B). Sensitivity was also lower for "deep jaundice of the trunk" as reported by PHWs (26-66%, Table 4A) and physicians (14-81%, Table 4B). Sensitivity of "deep jaundice of the head" was moderate for PHWs (32-90%, Table 4A) and physicians (53-97%, Table 4B). Sensitivity of physician assessments was particularly low across the various definitions for jaundice at La Paz (11-55%, Table 4B).

For peer review only

For peer review only - http://bmjopen.bmj.com/site/about/guidelines.xhtml

Table 4A. Sensitivity and specificity with 95% confidence intervals (in parentheses) of clinical signs of jaundice in neonates aged 1-20 days as assessed by primary healthcare workers for detecting levels of hyperbilirubinemia.

	Sensitivity						Specificity					
	Dhaka	Delhi	Chandigarh	Durban	Kumasi	La Paz	Dhaka	Delhi	Chandigarh	Durban	Kumasi	La Paz
**Serum bilirubin > 260 mmol/l (15 mg/dl)**												
**Deep jaundice of head**	(n=35) 51 (34,69)	(n=61) 90 (80,96)	(n=38) 32 (18, 49)	(n=18) 56 (31, 78)	(n=17)	(n=103) 75 (65, 83)	(n=41) 66 (49, 80)	(n=54) 48 (34,62)	(n=294) 89 (85, 92)	(n=103) 80 (71, 87)	(n=46)	(n=78) 49 (37, 60)
**Deep jaundice of trunk**	31 (17,49)	66 (52,77)	26 (13, 43)	47 (21, 73)		31 (22, 41)	78 (62, 89)	81 (69,91)	96 (93, 98)	82 (72, 90)		83 (73, 91)
**Any jaundice of distal extremities**	66 (48,81)	64 (51,76)	55 (38, 71)	56 (31, 78)	88 (64, 99)	42 (32, 52)	66 (49, 80)	80 (66,89)	89 (85, 92)	56 (46, 66)	76 (61, 87)	71 (59, 80)
**Any jaundice of distal extremities OR Deep jaundice of trunk**	66 (48,81)	77 (65,87)	58 (41, 74)	67 (41, 87)		44 (34, 54)	66 (49, 80)	72 (58,84)	88 (84, 92)	49 (39, 59)		69 (58, 79)
**Any jaundice of distal extremities OR Deep jaundice of head or trunk**	69 (51,83)	93 (84,98)	58 (41, 74)	83 (59, 96)		80 (71, 87)	63 (47, 78)	48 (34,62)	84 (79, 88)	48 (38, 58)		46 (35, 58)
**Serum bilirubin > 340 mmol/l (20 mg/dl)**												
**Deep jaundice of head**	(n=14) 79 (49,95)	(n=25) 96 (80,100)	(n=9) 67 (30, 93)	(n=2) *	(n=7)	(n=35) 94 (81, 99)	(n=62) 66 (53, 78)	(n=90) 34 (25,45)	(n=323) 88 (84, 92)	(n=119) 76 (67, 83)	(n=56)	(n=146) 42 (34, 51)
**Deep jaundice of trunk**	64 (35,87)	76 (55, 91)	44 (14, 79)			46 (29, 63)	82 (70, 91)	66 (55,75)	94 (91, 97)	77 (68, 85)		80 (73, 86)
**Any jaundice of distal extremities**	93 (66,100)	80 (59, 93)	89 (52, 100)		100 (59, 100)	57 (39, 74)	61 (48, 73)	67 (56,76)	86 (81, 89)	55 (45, 64)	66 (52, 78)	68 (60, 76)
**Any jaundice of distal extremities OR Deep jaundice of head or trunk**	93 (66,100)	88 (69, 97)	89 (52, 100)			60 (42, 76)	61 (48, 73)	56 (45,66)	85 (80, 89)	46 (37, 56)		67 (59, 75)
**Any jaundice of distal extremities OR Deep jaundice of head or trunk**	93 (66,100)	100 (86,100)	89 (52, 100)			97 (85, 100)	58 (45, 70)	33 (24,44)	81 (76, 85)	44 (35, 53)		38 (30, 47)

*Both cases were found to have deep jaundice of the face, and one also had jaundice of palms and soles.

For peer review only - http://bmjopen.bmj.com/site/about/guidelines.xhtml

Table 4B. Sensitivity and specificity with 95% confidence intervals (in parentheses) of clinical signs of jaundice in neonates aged 1-20 days as assessed by physicians for detecting levels of hyperbilirubinemia.

	Sensitivity						Specificity					
	Dhaka	Delhi	Chandigarh	Durban	Kumasi	La Paz	Dhaka	Delhi	Chandigarh	Durban	Kumasi	La Paz
**Serum bilirubin > 260 mmol/l (15 mg/dl)**												
**Deep jaundice of head**	(n=36) 53 (35, 70)	(n=61) 97 (89,100)	(n=39) 85 (69, 94)	(n=19) 79 (54, 94)	(n=17)	(n=103) 55 (45, 65)	(n=46) 78 (64, 89)	(n=57) 37 (24,51)	(n=340) 50 (44, 55)	(n=113) 88 (80, 93)	(n=53)	(n=76) 89 (80, 95)
**Deep jaundice of trunk**	19 (8, 36)	81 (69, 90)	79 (64, 91)	44 (20, 70)		14 (8, 23)	96 (85, 99)	81 (68,90)	92 (89, 95)	90 (82, 95)		100 (95,100)
**Any jaundice of distal extremities**	56 (38, 72)	66 (52, 77)	38 (23, 55)	42 (20, 67)	59 (33, 82)	11 (5, 18)	80 (66, 91)	95 (85,99)	97 (94, 98)	93 (87, 97)	74 (60, 85)	99 (93,100)
**Any jaundice of distal extremities OR Deep jaundice of trunk**	56 (38, 72)	90 (80, 96)	79 (64, 91)	47 (24, 71)		15 (9, 24)	80 (66, 91)	79 (66,89)	91 (88, 94)	87 (79, 92)		99 (93,100)
**Any jaundice of distal extremities OR Deep jaundice of head or trunk**	64 (46, 79)	98 (91,100)	85 (69, 94)	79 (54, 94)		55 (45, 65)	74 (59, 86)	37 (24,51)	49 (44, 55)	83 (75, 90)		88 (79, 94)
**Serum bilirubin > 340 mmol/l (20 mg/dl)**												
**Deep jaundice of head**	(n=14) 79 (49, 95)	(n=26) 100 (87,100)	(n=9) 100 (66, 100)	(n=2) *	(n=7)	(n=36) 81 (64, 92)	(n=68) 74 (61, 83)	(n=92) 25 (16,35)	(n=370) 47 (42, 53)	(n=130) 79 (71, 86)	(n=63)	(n=143) 75 (67, 82)
**Deep jaundice of trunk**	50 (23, 77)	92 (75, 99)	100 (66, 100)			28 (14, 45)	97 (90, 100)	60 (50,70)	87 (83, 90)	85 (77, 91)		97 (92, 99)
**Any jaundice of distal extremities**	86 (57, 98)	85 (65, 96)	78 (40, 97)		71 (29, 96)	17 (6, 33)	75 (63, 85)	77 (67,85)	95 (92, 97)	88 (82, 93)	70 (57, 81)	96 (91, 98)
**Any jaundice of distal extremities OR Deep jaundice of head or trunk**	86 (57, 98)	100 (87,100)	100 (66, 100)			28 (14, 45)	75 (63, 85)	55 (44,65)	86 (82, 90)	82 (75, 88)		95 (90, 98)
**Any jaundice of distal extremities OR Deep jaundice of head or trunk**	93 (66,100)	100 (87,100)	100 (66, 100)			81 (64, 92)	68 (55, 78)	24 (15,34)	47 (42, 52)	75 (67, 83)		74 (66, 81)

*Both cases were found to have deep jaundice of the face, and one also had jaundice of palms and soles.

For peer review only - http://bmjopen.bmj.com/site/about/guidelines.xhtml

Specificity of PHW assessments (Table 4A) was highest for "deep jaundice of the trunk" (78-96%) and ranged from 48-89% across all other jaundice algorithms at all study locations. Specificity varied across sites for the algorithm "any jaundice of the distal extremities or deep jaundice of the trunk or head" (46-84%). Specificity of assessments by physicians (Table 4B) was relatively high across all algorithms at all sites (74-100%) except at the Indian sites for "deep jaundice of the head" and "any jaundice of the distal extremities or deep jaundice of the trunk or head" (37-50%).

For the higher threshold of hyperbilirubinemia (>340 μmol/L or >20 mg/dL), sensitivity was higher and specificity was comparable to slightly lower compared to the findings at the lower serum bilirubin threshold for PHWs (Table 4A) and physicians (Table 4B). High sensitivity was found for "any jaundice of the distal extremities or deep jaundice of the trunk or head" for PHWs (89-100%, Table 4A) and physicians (81-100%, Table 4B). Specificity for this algorithm was more variable for both PHWs (33-81%, Table 4A) and physicians (24-75%, Table 4B). A reasonable balance of sensitivity (71-100%) and specificity (55-95%) across both PHW and physician assessments was observed, excluding La Paz, for the definition "any jaundice of distal extremities."

Agreement between primary healthcare workers and physicians in assessment of jaundice

Agreement between PHWs and physicians was variable for specific definitions of jaundice and across sites (Table 5). Agreement between PHWs and physicians in identifying neonates with any signs of jaundice on the head was generally good but agreement for depth of jaundice and

For peer review only - http://bmjopen.bmj.com/site/about/guidelines.xhtml

for jaundice of the extremities was variable. Overall agreement was highest for Delhi (0.68-0.82 across jaundice algorithms).

Table 5. Agreement between primary healthcare workers and physicians in detecting clinical signs of jaundice in neonates aged 1-20 days, by study site.

	Study location					
	Dhaka	Delhi	Chandigarh	Durban	Kumasi	La Paz
Deep jaundice of the head	488<br>0.14 0.12<br>0.77	334<br>0.27 0.31<br>0.82	703<br>0.06 0.3<br>0.23	414<br>0.09 0.07<br>0.5		293<br>0.43 0.24<br>0.42
Deep jaundice of the trunk (chest or abdomen)	488<br>0.08 0.02<br>0.43	334<br>0.15 0.19<br>0.73	703<br>0.03 0.08<br>0.35	323<br>0.08 0.05<br>0.61		293<br>0.16 0.05<br>0.44
Any jaundice of the distal extremities (palms or soles)	488<br>0.15 0.10<br>0.61	333<br>0.16 0.13<br>0.68	703<br>0.08 0.04<br>0.41	414<br>0.17 0.04<br>0.29	456<br>0.16 0.12<br>0.49	292<br>0.24 0.04<br>0.21

Data for each definition of clinical jaundice are displayed as follows: (row 1) number of infants evaluated by both observers; (row 2) proportion of infants judged positive by primary healthcare workers and by physicians, respectively; (row 3) kappa statistic for measuring agreement beyond chance.

Discussion

The study is the largest evaluation reported to date on the validity of clinical assessments for jaundice by PHWs and physicians in identifying neonates with hyperbilirubinemia at levels indicating need for referral-level care. We observed that a finding of “any jaundice of the distal extremities” (palms or soles) by PHWs or physicians had sensitivity and specificity generally exceeding about 70% across locations for detecting neonates aged 1-20 days with bilirubin levels >340 µmol /L (20 mg/dL) – a level that requires acute intervention in a health facility, including evaluation for plasma exchange transfusion in at-risk and preterm babies. Given that this level of jaundice places infants at risk of severe morbidity, we also note that the broader algorithm “any jaundice of the distal extremities or deep jaundice of the trunk or head” had high sensitivity for

For peer review only

For peer review only - http://bmjopen.bmj.com/site/about/guidelines.xhtml

both PHWs (89-100%) and physicians (81-100%), although with variable specificity across study sites (33-81% for PHWs, 24-75% for physicians). Use of this latter algorithm could result in the referral of more at-risk infants (based on high sensitivity), but also, depending on specificity at the site, more infants without significant hyperbilirubinemia. This latter algorithm may be preferable in settings in which prompt referral and access to timely measurement and management of hyperbilirubinemia are lacking or anticipated to be delayed. Clinical signs were also discriminatory, but less so, for identifying infants who had moderately elevated bilirubin (>260 µmol/L). For this lower threshold level indicating need for phototherapy, the algorithm "any jaundice of the distal extremities or deep jaundice of the trunk or head" also had the highest overall sensitivity across sites (58-93% for PHWs and 55-98% for physicians). Thus, in settings where it is not possible to measure serum bilirubin, such as first level health facilities in LMICs which use IMCI, it is recommended to refer neonates aged 1-20 days with yellow palms and soles or deep jaundice on the trunk or head to a hospital for further evaluation and management.

We found very few cases in which jaundice appeared on the first day after delivery or lasted beyond three weeks of age; all these babies should be referred to a health facility for further assessment and management, including direct and total bilirubin measurement. A small percentage of infants aged 21-59 days had jaundice (n=96, 3.7%); jaundice presenting in this age group is presumed pathological and all such infants should also be referred for further clinical and laboratory workup.

Several previous studies have assessed the value of clinical signs for predicting high levels of serum bilirubin in neonates.[10-19] In many LMICs where births commonly occur either at home or

For peer review only - http://bmjopen.bmj.com/site/about/guidelines.xhtml

at peripheral health clinics, laboratory measurement of serum bilirubin may not be easily available and clinical signs have to be used as a basis for referral. Similar to some previous studies in LMICs, we found that serum bilirubin levels were generally higher in neonates with jaundice of more caudal parts of the body.[12-16] Jaundice extending most distally to the palms and soles was most discriminatory in identifying neonates with very high levels of bilirubin in need of urgent evaluation for treatment, and thus, has clinical relevance. However, jaundice that deeply involved the trunk or head was also useful clinically and recognition of this less distal but more sensitive sign, as part of the algorithm "any jaundice of the distal extremities or deep jaundice of the trunk or head" may be particularly important in low resource settings with fewer diagnostic and treatment capabilities.

In general, PHWs had higher sensitivity than physicians in identifying neonates with hyperbilirubinemia based on identification of clinical signs of jaundice. Overall, the sensitivity of any jaundice on palms and soles in detecting infants with high bilirubin levels >340 μmol /L was higher for PHWs than for physicians, but the specificity was about 10 percentage points lower in most locations. For identifying infants with bilirubin levels >260 μmol /L, specificity was slightly higher for PHWs, while sensitivity was similar for both cadres. These results emphasise that once neonates with suspected hyperbilirubinemia based on PHW clinical asserssment reach a health facility, further physician clinical assessment and laboratory evaluations must be performed whenever possible to determine need for treatment. We observed considerable variation in the performance of clinical signs of jaundice between study locations, despite attempting to standardise PHW training and physician supervision. Overall, clinical signs had lower sensitivity in La Paz and lower specificity in Dhaka and Durban, and agreement for the

For peer review only - http://bmjopen.bmj.com/site/about/guidelines.xhtml

For peer review only

presence of jaundice on the palms and soles was lowest in Durban and La Paz compared to the other locations. These findings highlight the importance of local evaluation and adaptations of algorithms.

Variable skin pigmentation is an important potential confounding factor in the clinical detection of jaundice.[13] In Kumasi, where skin pigmentation was darkest among the study locations, study investigators were not confident that they could identify jaundice on the face, chest or thighs; as a result, their assessments of jaundice were limited to the sclerae, gums, palms and soles. Nevertheless, PHWs in Kumasi were able to identify neonates needing referral for hyperbilirubinemia based on recognition of jaundice on the palms and soles, with good sensitivity and reasonable specificity.

This study had several strengths including its size (n=1242) and multiple sites, jaundice assessment at several locations on each infant, and systematic evaluation of PHW assessments of jaundice. The findings have the potential to be generalised to large populations in LMICs.

This study had several limitations. We did not attempt to objectively assess the depth of jaundice, for example by the use of color scales for comparison. Also, due to inconsistencies in obtaining blood samples for bilirubin measurement, 22% of infants with some degree of jaundice did not have laboratory work done. In addition, the decision to draw blood for bilirubin detection was based on the study physicians' diagnoses and not the PHWs' assessments. Consequently, some infants with jaundice identified by PHWs did not have blood samples obtained. Since the study design focused on sick young infants presenting for care, asymptomatic infants with

For peer review only - http://bmjopen.bmj.com/site/about/guidelines.xhtml

jaundice may not have been brought for assessment, and thus, the sensitivity and specificity of clinical assessments may vary at population level and warrant further investigation. However, infants presenting for routine health care (e.g., well baby checks) may also be at risk for complications of jaundice and therefore should be screened. Finally, this study did not include sufficient numbers of preterm infants to allow for independent assessment of the utility of jaundice detection in this subgroup of infants, who are especially vulnerable to morbidity and mortality from hyperbilirubinemia. It is also challenging to accurately identify gestational age in LMICs where ultrasound assessment may not be available. Therefore, a more conservative approach to referral of jaundiced infants suspected to be preterm is advisable, for example use of the algorithm "any jaundice of the distal extremities or deep jaundice of the trunk or head."

Conclusion

Further standardisation of training to ensure greater consistency among PHWs' assessments, as well as local research and monitoring in settings where IMCI guidelines are implemented would be useful to further refine the assessment and referral criteria for jaundice. The current IMCI algorithm focuses on jaundice of palms and soles as indicative of need for urgent referral. However, the more broadly defined algorithm "any jaundice of the distal extremities or deep jaundice of the trunk or head" may identify more at-risk neonates – although at a potential cost of referral of greater numbers of infants not needing acute intervention (e.g., phototherapy, exchange transfusion) – and may be preferred in settings where delay in receiving care is anticipated. Local evidence-based adaptations of these algorithms are needed, particularly regarding consideration of deep staining of the trunk or head and the balance between increased sensitivity, enabling the detection of greater numbers of infants needing further assessment, and

For peer review only - http://bmjopen.bmj.com/site/about/guidelines.xhtml

increased specificity and targeting of scare diagnostic and treatment resources. Many hospitals in LMICs cannot determine bilirubin levels reliably.[29] In addition to building basic laboratory capacity in developing country hospitals, further refinement and evaluation of the utility of point-of-care technologies[20-22,30] to augment clinical assessment for detection of hyperbilirubinemia is warranted.

Acknowledgements

We thank the local communities at the study sites for their participation in the study, and also thank all members of the Young Infant Clinical Signs Study Group (YICSSG) who made this study possible.

YICSSG members include (exclusive of named co-authors):

Study sites: Bangladesh: *Clinical investigators:* A. S. M. Nawshad Uddin Ahmed, Md. Monir Hossain; *Study physicians:* Nazmun Nahar; *Nurses*: Amala Baidya, Mahmuda Parul; *Laboratory personnel*: Maksuda Islam, Tania Nasreen; *Data management*: Md. Rezaur Rahaman; **Bolivia**: *Study physicians*: Teresa Villagomez, Pablo Mattos; Manuel Pantoja Ludueña, Remedios Zumarán; *Study nurses*: Irma Quispe, Willy Tarqui, Lourdes Checa, Claudia Canqui; *Data management:* Erick Dueñas, Omar Vargas; **Ghana**: *Study physicians:*G. Plange-Rhule, Osei Akoto; *Laboratory:* M. Lartey; *Data management:* Henrietta Akpene; **India, Chandigarh:** *Clinical investigators:* Rupinder Narang; *Study physicians:* Prasad Muley, Satish Misra; *Nurses:* Tapasaya, Sanjay Rani; *Laboratory:* Pallab Ray, Tamanna Gaur; *Data management:* Vishal Kanojia, Ajay Dogra; **India, Delhi:** *Clinical investigators:* Harish Chellani, M. S. Prasad; *Study physician:* A. Satyavani; *Nurses:* Jyoti, Raji John; *Laboratory:* Arti Kapil; *Data management:*

For peer review only - http://bmjopen.bmj.com/site/about/guidelines.xhtml

Sanjeev Negi, Narinder Singhal**; South Africa:** *Clinical investigator:* Mirian Adhikari; *Nurse:* Sister Mojaphelo; *Laboratory:* Wim Sturm; *Data management:* Precious Sikhakhane.

Study advisors: Kim Mulholland, Vinod Paul, Eric Simoes, Jelka Zupan

Data Management: Philip Greenwood, Claudine Chionh, Murdoch Children's Research Institute.

For peer review only

For peer review only - http://bmjopen.bmj.com/site/about/guidelines.xhtml

References

1. Young Infants Clinical Signs Study Group (YICSSG). Clinical signs predicting severe illness in young infants: a multicentre study. Lancet 2008;371:135-42.

2. Cashore WJ. The neurotoxicity of bilirubin. Clin Perinatol 1990;17:437-47.

3. Ip S, Chung M, Kulig J, O'Brien R, Sege R, Glicken S, et al. American Academy of Pediatric Technical Report. An evidence-based review of important issues concerning neonatal hyperbilirubinemia. Pediatrics 2004;114:e130-153.

3. Totapally BR, Torbati D. Neonatal jaundice. Int Pediatr 2005;20:47-54.

5. Maulik PK, Darmstadt GL. Childhood disability in low and middle income countries: overview of screening, prevention, services, legislation and epidemiology. Pediatrics 2007;120(Suppl 1);S1-S55.

6. Bhutani VK, Zipursky A, Blencowe H, Khanna R, Sgro M, Ebbesen F, et al. Neonatal hyperbilirubinemia and Rhesus disease of the newborn: incidence and impairment estimates for 2010 at regional and global levels. Pediatr Res 2013;74:86-100.

7. Darmstadt GL, Baqui AH, Choi Y, Bari S, Rahman SM, Mannan I, et al. Validation of community health workers' assessment of neonatal illness in rural Bangladesh. Bull World Health Organ 2009;87:12-19.

8. World Health Organization WH. IMCI chart booklet. World Health Organization, Geneva, Switzerland. 2014.

9. Boschi-Pinto C, Labadie G, Dilip TR, Oliphant N, Dalglish SL, Aboubaker S, et al. Global implementation survey of Integrated Management of Childhood Illness (IMCI): 20 years on. BMJ Open. 2018;8(7):e019079.

For peer review only

10. Goswami V, Dutta AK, Singh V, Chandra J. Evaluation of simple clinical signs of illness in young infants (0-2 months) and its correlation with WHO IMCI algorithm (7 days to 2 months). Indian Pediatr 2006;43:1042-1049.

11. Hatzenbuehler L, Zaidi AK, Sundar S, Sultana S, Abbasi F, A Rizvi, Darmstadt GL. Validity of neonatal jaundice evaluation by primary health-care workers and physicians in Karachi, Pakistan. J Perinatol 2010;30(9):616-.

12. Ebbesen F. Relationship between the cepahlo-pedal progress of clinical icterus and the serum bilirubin concentration in newborn infants without blood type sensitization. Acta Obstet Gynecol Scand 1975;54:329-332.

13. Knudsen A, Brodersen R. Skin colour and bilirubinemia in neonates. Arch Dis Child 1989;64:605-9.

14. Madlon-Kay DJ. Recognition of the presence and severity of newborn jaundice by parents, nurses, physicians, and icterometer. Pediatrics 1997;100(3):E3.

15. Madlon-Kay DJ. Home health nurse clinical assessment of neonatal jaundice: comparison of three methods. Arch Pediatr Adolesc Med 2001;155:583-6.

16. Riskin A, Kuglman A, Abend-Weinger M, Green M, Hemo M, Bader D. In the eye of the beholder: how accurate is clinical estimation of jaundice in newborns? Acta Paediatr 2003;92:574-576.

17. Kramer LI. Advancement of dermal icterus in the jaundiced newborn. Amer J Dis Child 1969;118:454-458.

18. Moyer VA, Ahn C, Sneed S. Accuracy of clinical judgement in neonatal jaundice. Arch Pediatr Adolesc Med 2000;154:391-4.

For peer review only - http://bmjopen.bmj.com/site/about/guidelines.xhtml

19. Tayaba R, Gribetz D, Gribetz I, Holzman IR. Noninvasive estimation of serum bilirubin. Pediatrics 1998;102(3):E28.

20. Boo NY, Chang YF, Leong YX, Tok ZY, Hooi LC, Che SC, Latif ZA. The point-of-care Bilistick method has very short turn-around-time and high accuracy at lower cutoff levels to predict laboratory-measured TSB. Pediatr Res 2019 Aug;86(2):216-220. doi: 10.1038/s41390-019-0304-0.

21. Rohsiswatmo R, Oswari H, Amandito R, Sjakti HA, Windiastuti E, Roeslani RD, Barchia I. Agreement test of transcutaneous bilirubin and bilistick with serum bilirubin in preterm infants receiving phototherapy BMC Pediatr 2018 Sep 29;18(1):315. doi: 10.1186/s12887-018-1290-9.

22. Greco C, Iskander IF, Houchi SZE, Rohsiswatmo R, Rundjan L, Ogala WN, et al. Diagnostic performance analysis of the point-of-care Bilistick system in identifying severe neonatal hyperbilirubinemia by a multi-country approach. EClinicalMedicine 2018 Jul 17;1:14-20. doi: 10.1016/j.eclinm.2018.06.003.

23. Deorari AK, Chellani H, Carlin JB, Greenwood P, Prasad MS, Satyavani A, et al. Clinicoepidemiological profile and predictors of severe illness in young infants (<60 days) reporting to a hospital in North India. Indian Pediatr 2007;44:739-748.

24. Narang A, Kumar P, Narang R, Ray P, Carlin JB, Greenwood P, et al. Clinico-epidemiological profile and validation of symptoms and signs of severe illness in young infants (<60 days) reporting to a district hospital in North India. Indian Pediatr 2007;44:751-759.

For peer review only - http://bmjopen.bmj.com/site/about/guidelines.xhtml

25. Yeboah-Antwi K, Addo-Yobo E, Adu-Sarkodie Y, Carlin JB, Plange-Rhule G, Osei Akoto A, et al. Clinico-epidemiological profile and predictors of severe illness in young infants (0-59 days) in Ghana. Ann Trop Paediatr 2008;28:35-43.

26. Jeena PM, Adhikari M, Carlin JB, Qazi S, Weber MW, Hamer DH. Clinical profile and predictors of severe illness in young South African infants (<60 days). S Afr Med J. 2008;98(11):883-888.

27. WHO Young Infants Study Group. Methodology for a multicenter study of serious infections in young infants in developing countries. Pediatric Infectious Diseases Journal 1999;10 (Suppl.):S8-S16.

28. WHO. Pocket book of hospital care for children: Guidelines for the management of common childhood illnesses, second edition. WHO Geneva 2013, ISBN: 978 92 4 154837 3.

29. Nolan T, Angos P, Cunha AJ, Muhe L, Qazi S, Simoes EA, et al. Quality of hospital care for seriously ill children in developing countries. Lancet 2001;357(9250):106-10.

30. Lee AC, Folger LV, Rahman M, et al. A novel icterometer for hyperbilirubinemia screening in low-resource settings. Pediatrics. 2019;143(5):e20182039.

For peer review only

Author Contributions

GLD, DHH, JBC and MWW co-coordinated study design, and provided oversight to overall implementation, data collection, and data analysis. Site PIs coordinated implementation and data collection at Chandigarh (PK, AN, PR), Delhi (AD), Kumasi (EAY, YAS, KYA), Durban (PMJ), Bangladesh (MAKAC, SKS), and La Paz (AB and EM). RB contributed to data analysis and interpretation of data. GLD wrote the first draft of the manuscript. All authors reviewed and provided input to the manuscript and approved the paper for publication.

Competing interests

RB and MWW are WHO staff members. MWW contributed to study design, implementation, data analysis and manuscript writing and RB contributed to data analysis and manuscript writing. The opinions expressed in this paper do not necessarily represent the position of the WHO. The funding agencies did not influence the conduct or outcomes of the study, data analysis or interpretation, or preparation of this paper. GLD, DHH, JBC, and MWW had access to raw data. The corresponding author had full access to all data and had final responsibility for the decision to submit the paper for publication.The authors declare no other potential competing interests.

Patient consent for publication

Not required.

Provenance and peer review

Not commissioned; externally peer reviewed.

Figure legends

Figure 1. Box plots of serum bilirubin levels in neonates aged 1-20 days according to clinical signs of jaundice detected by physicians, by study location. Horizontal lines indicate the two thresholds for hyperbilirubinemia used in subsequent analysis (260 and 340 umol/L).

For peer review only

For peer review only - http://bmjopen.bmj.com/site/about/guidelines.xhtml

Figure 1. Box plots of serum bilirubin levels in neonates aged 1-20 days according to clinical signs of jaundice detected by physicians, by study location. Horizontal lines indicate the two thresholds for hyperbilirubinemia used in subsequent analysis (260 and 340 umol/L).

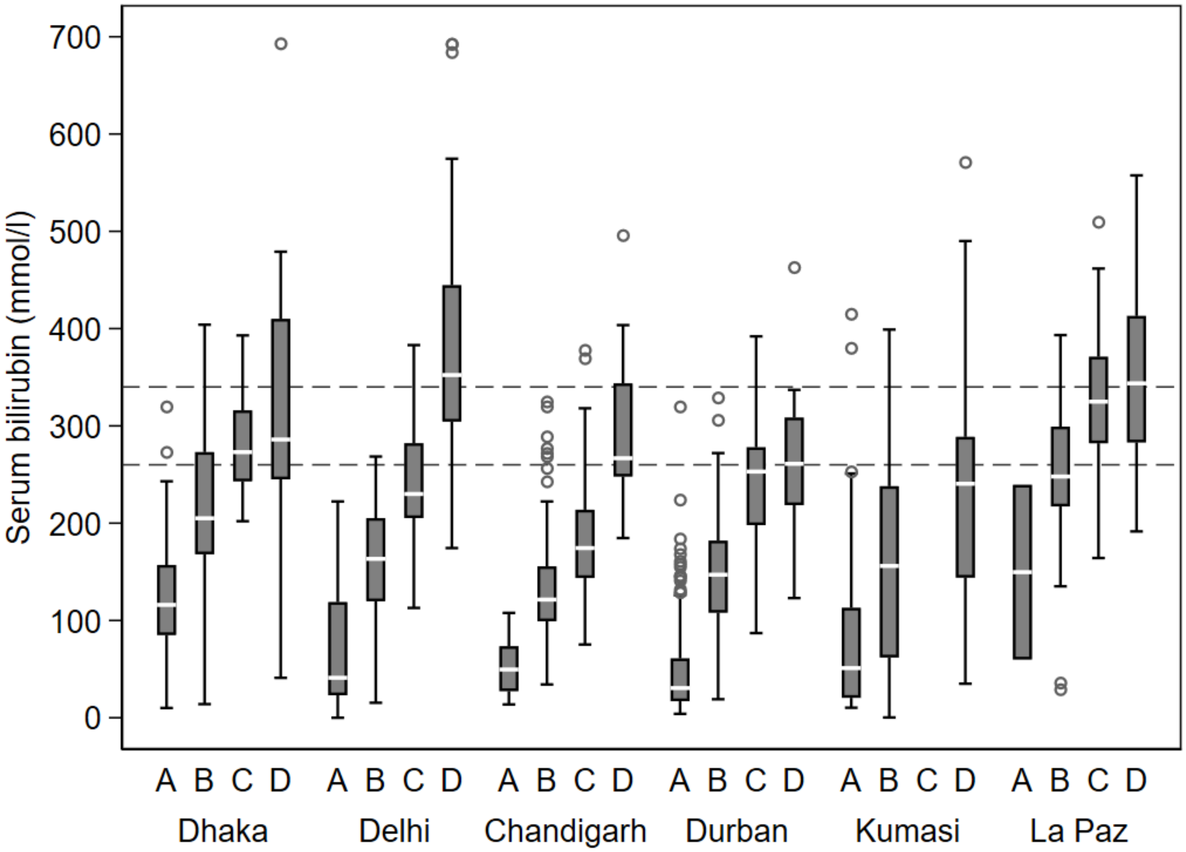

For peer review only - http://bmjopen.bmj.com/site/about/guidelines.xhtml

STROBE Statement—checklist of items that should be included in reports of observational studies

	Item No	Recommendation	
**Title and abstract**	1	(*a*) Indicate the study's design with a commonly used term in the title or the abstract	Title, p. 1
		(*b*) Provide in the abstract an informative and balanced summary of what was done and what was found	Abstract, p. 3-4
**Introduction**			
Background/rationale	2	Explain the scientific background and rationale for the investigation being reported	Introduction, pp. 5-6
Objectives	3	State specific objectives, including any prespecified hypotheses	Introduction, p. 6
**Methods**			
Study design	4	Present key elements of study design early in the paper	Methods, pp. 6-9
Setting	5	Describe the setting, locations, and relevant dates, including periods of recruitment, exposure, follow-up, and data collection	Methods, pp. 6-9, Table 1
Participants	6	(*a*) *Cohort study*—Give the eligibility criteria, and the sources and methods of selection of participants. Describe methods of follow-up<br>*Case-control study*—Give the eligibility criteria, and the sources and methods of case ascertainment and control selection. Give the rationale for the choice of cases and controls<br>*Cross-sectional study*—Give the eligibility criteria, and the sources and methods of selection of participants	Methods, pp. 6-7
		(*b*) *Cohort study*—For matched studies, give matching criteria and number of exposed and unexposed<br>*Case-control study*—For matched studies, give matching criteria and the number of controls per case	N/A
Variables	7	Clearly define all outcomes, exposures, predictors, potential confounders, and effect modifiers. Give diagnostic criteria, if applicable	Methods, pp. 8-9
Data sources/ measurement	8*	For each variable of interest, give sources of data and details of methods of assessment (measurement). Describe comparability of assessment methods if there is more than one group	Methods, pp. 8-9
Bias	9	Describe any efforts to address potential sources of bias	Methods, p. 8
Study size	10	Explain how the study size was arrived at	Methods, p. 6

For peer review only - http://bmjopen.bmj.com/site/about/guidelines.xhtml

Quantitative variables	11	Explain how quantitative variables were handled in the analyses. If applicable, describe which groupings were chosen and why	Methods, pp. 9-10
Statistical methods	12	(*a*) Describe all statistical methods, including those used to control for confounding	Methods, pp. 9-10
		(*b*) Describe any methods used to examine subgroups and interactions	N/A
		(*c*) Explain how missing data were addressed	pp. 9-10
		(*d*) *Cohort study*—If applicable, explain how loss to follow-up was addressed<br>*Case-control study*—If applicable, explain how matching of cases and controls was addressed<br>*Cross-sectional study*—If applicable, describe analytical methods taking account of sampling strategy	N/A
		(*e*) Describe any sensitivity analyses	N/A

Continued on next page

For peer review only

**Results**			
Participants	13*	(a) Report numbers of individuals at each stage of study—eg numbers potentially eligible, examined for eligibility, confirmed eligible, included in the study, completing follow-up, and analysed	Results, p. 12, Tables 2-5
		(b) Give reasons for non-participation at each stage	N/A
		(c) Consider use of a flow diagram	Summarized p. 12, Table 2
Descriptive data	14*	(a) Give characteristics of study participants (eg demographic, clinical, social) and information on exposures and potential confounders	See parent study, reference 1
		(b) Indicate number of participants with missing data for each variable of interest	Results, Tables 2-5
		(c) *Cohort study*—Summarise follow-up time (eg, average and total amount)	Methods, p. 9
Outcome data	15*	*Cohort study*—Report numbers of outcome events or summary measures over time	Tables 2-5
		Case-control study—Report numbers in each exposure category, or summary measures of exposure	N/A
		Cross-sectional study—Report numbers of outcome events or summary measures	N/A
Main results	16	(*a*) Give unadjusted estimates and, if applicable, confounder-adjusted estimates and their precision (eg, 95% confidence interval). Make clear which confounders were adjusted for and why they were included	N/A
		(*b*) Report category boundaries when continuous variables were categorized	Results, pp. 13-18
		(*c*) If relevant, consider translating estimates of relative risk into absolute risk for a meaningful time period	N/A
Other analyses	17	Report other analyses done—eg analyses of subgroups and interactions, and sensitivity analyses	N/A
**Discussion**			
Key results	18	Summarise key results with reference to study objectives	Discussion, pp. 13-18
Limitations	19	Discuss limitations of the study, taking into account sources of potential bias or imprecision. Discuss both direction and magnitude of any potential bias	Discussion, p. 21-22
Interpretation	20	Give a cautious overall interpretation of results considering objectives, limitations, multiplicity of analyses, results from similar studies, and other relevant evidence	Discussion, pp. 18-23
Generalisability	21	Discuss the generalisability (external validity) of the study results	Discussion, pp. 18-23; Conclusion, pp. 22-23
**Other information**			
Funding	22	Give the source of funding and the role of the funders for the present study and, if applicable, for the original study on which the present article is based	p. 11

For peer review only - http://bmjopen.bmj.com/site/about/guidelines.xhtml

*Give information separately for cases and controls in case-control studies and, if applicable, for exposed and unexposed groups in cohort and cross-sectional studies.

Note: An Explanation and Elaboration article discusses each checklist item and gives methodological background and published examples of transparent reporting. The STROBE checklist is best used in conjunction with this article (freely available on the Web sites of PLoS Medicine at http://www.plosmedicine.org/, Annals of Internal Medicine at http://www.annals.org/, and Epidemiology at http://www.epidem.com/). Information on the STROBE Initiative is available at www.strobe-statement.org.

For peer review only

BMJ Open

Validation of visual estimation of neonatal jaundice in low- and middle-income countries: a multicentre observational cohort study

Journal:	*BMJ Open*
Manuscript ID	bmjopen-2020-048145.R1
Article Type:	Original research
Date Submitted by the Author:	22-Sep-2021
Complete List of Authors:	Darmstadt, Gary; Stanford University School of Medicine, Pediatrics<br>Hamer, D.; Boston University School of Public Health, Department of Global Health<br>Carlin, John; Murdoch Childrens Research Institute, Clinical Epidemiology and Biostatistics Unit<br>Jeena, Prakash; University of KwaZulu-Natal Nelson R Mandela School of Medicine, Department of Paediatrics & Child Health<br>Mazzi, Eduardo; Hospital del Nino Dr Ovidio Aliaga Uria<br>Narang, Anil; Post Graduate Institute of Medical Education and Research<br>Deorari, A; All India Institute of Medical Sciences, Pediatrics<br>Addo-Yobo, Emmanuel; Kwame Nkrumah University of Science and Technology, School of Medical Sciences<br>Chowdhury, MAK Azad; Dhaka Shishu Hospital, Neonatology<br>Kumar, Praveen; Post Graduate Institute of Medical Education and Research<br>Abu-Sarkodie, Yaw; Kwame Nkrumah University of Science and Technology<br>Yeboah-Antwi, Kojo; Kwame Nkrumah University of Science and Technology<br>Ray, Pallab; Post Graduate Institute of Medical Education and Research<br>Bartos, Andres; Hospital Materno-Infantil, Pediatrics<br>Saha, Samir K.; Child Health Research Foundation<br>Foote, Eric; Stanford University School of Medicine, Pediatrics<br>Bahl, Rajiv; World Health Organization, Department of Maternal, Newborn, Child and Adolescent Health<br>Weber, Martin; World Health Organization Regional Office for Europe
**Primary Subject Heading**:	Global health
Secondary Subject Heading:	Paediatrics, Public health
Keywords:	NEONATOLOGY, Community child health < PAEDIATRICS, PUBLIC HEALTH, PRIMARY CARE, Epidemiology < TROPICAL MEDICINE

I, the Submitting Author has the right to grant and does grant on behalf of all authors of the Work (as defined in the below author licence), an exclusive licence and/or a non-exclusive licence for contributions from authors who are: i) UK Crown employees; ii) where BMJ has agreed a CC-BY licence shall apply, and/or iii) in accordance with the terms applicable for US Federal Government officers or employees acting as part of their official duties; on a worldwide, perpetual, irrevocable, royalty-free basis to BMJ Publishing Group Ltd ("BMJ") its licensees and where the relevant Journal is co-owned by BMJ to the co-owners of the Journal, to publish the Work in this journal and any other BMJ products and to exploit all rights, as set out in our licence.

The Submitting Author accepts and understands that any supply made under these terms is made by BMJ to the Submitting Author unless you are acting as an employee on behalf of your employer or a postgraduate student of an affiliated institution which is paying any applicable article publishing charge ("APC") for Open Access articles. Where the Submitting Author wishes to make the Work available on an Open Access basis (and intends to pay the relevant APC), the terms of reuse of such Open Access shall be governed by a Creative Commons licence – details of these licences and which Creative Commons licence will apply to this Work are set out in our licence referred to above.

Other than as permitted in any relevant BMJ Author's Self Archiving Policies, I confirm this Work has not been accepted for publication elsewhere, is not being considered for publication elsewhere and does not duplicate material already published. I confirm all authors consent to publication of this Work and authorise the granting of this licence.

For peer review only

Validation of visual estimation of neonatal jaundice in low-and middle-income countries: a multicentre observational cohort study

Running title: Multicentre clinical assessment of neonatal jaundice

Gary L. Darmstadt[1], Davidson H. Hamer[2,3], John B. Carlin[4], Prakash M. Jeena[5], Eduardo Mazzi[6], Anil Narang[7], Ashok Deorari[8], Emmanuel Addo-Yobo[9], MAK Azad Chowdury[10], Praveen Kumar, DM[7], Yaw Adu-Sarkodie[9], Kojo Yeboah-Antwi[9], Pallab Ray[7], Andrés Bartos[11], Samir K. Saha[12], Eric M. Foote[1], Rajiv Bahl[13], Martin W. Weber[14], for the Young Infants Clinical Signs Study (YICSS) Group

1. Department of Pediatrics, Stanford University School of Medicine, Stanford, CA, USA;
2. Department of Global Health, Boston University School of Public Health, Boston, MA, USA
3. Section of Infectious Diseases, Department of Medicine, Boston University School of Medicine, Boston, MA, USA
4. Clinical Epidemiology and Biostatistics Unit, Murdoch Children's Research Institute and The University of Melbourne, Melbourne, Australia
5. Department of Paediatrics & Child Health, Nelson R. Mandela School of Medicine, University of KwaZulu-Natal, Durban, South Africa
6. Department of Pediatrics, Hospital del Niño "Dr. Ovidio Aliaga Uría", La Paz, Bolivia
7. Post Graduate Institute of Medical Education and Research, Chandigarh, India
8. Department of Pediatrics, Division of Neonatology, All India Institute for Medical Sciences, Delhi, India

For peer review only

9. School of Medical Sciences, Kwame Nkrumah University of Science & Technology, Kumasi, Ghana

10. Department of Neonatology, Dhaka Shishu Hospital, Dhaka, Bangladesh

11. Department of Pediatrics, Hospital Materno Infantil, La Paz, Bolivia

12. Child Health Research Foundation, Dhaka, Bangladesh

13. Newborn Health Unit, World Health Organization, Geneva, Switzerland

14. World Health Organization Regional Office for Europe, Copenhagen, Denmark

[a] Previous affiliation: Department of International Health, Johns Hopkins School of Public Health, Baltimore, MD, USA

Correspondence: Gary L. Darmstadt, MD, MS, Department of Pediatrics, Stanford University School of Medicine, 1701 Page Mill Road, Palo Alto, CA 94304, USA, gdarmsta@stanford.edu, tel: 650-724-6014

Key words: Jaundice, algorithm, hyperbilirubinemia, neonatal morbidity, primary care

Words: Abstract, 300; text, 4046

Tables: 5; **Figures:** 1

For peer review only

Abstract

Objective: Determine the sensitivity and specificity of neonatal jaundice visual estimation by primary healthcare workers (PHWs) and physicians as predictors of hyperbilirubinemia.

Design: Multiccenter observational cohort study.

Setting: Hospitals in Chandigarh and Delhi, India; Dhaka, Bangladesh; Durban, South Africa; Kumasi, Ghana; La Paz, Bolivia.

Participants: Neonates aged 1-20 days (n=2642) who presented to hospitals for evaluation of acute illness. Infants referred for any reason from another health facility or those needing immediate cardiopulmonary resuscitation were excluded.

Outcome measures: Infants were evaluated for distribution (head, trunk, distal extremities) and degree (mild, moderate, severe) of jaundice by PHWs and physicians. Serum bilirubin level was determined for infants with jaundice, and analyses of sensitivity and specificity of visual estimations of jaundice utilised bilirubin thresholds of >260 μmol/L (need for phototherapy) and >340 μmol/L (need for emergency intervention in at-risk and preterm babies).

Results: 1241 (47.0%) neonates had jaundice. High sensitivity for detecting neonates with serum bilirubin >340 μmol/L was found for "any jaundice of the distal extremities (palms or soles) OR deep jaundice of the trunk or head" for both PHWs (89-100%) and physicians (81-100%) across study sites; specificity was more variable. "Any jaundice of the distal extremities" identified by PHWs and physicians had sensitivity of 71-100% and specificity of 55-95%, excluding La Paz. For the bilirubin threshold >260 μmol/L, "any jaundice of the distal extremities OR deep jaundice of the trunk or head" had the highest sensitivity across sites (PHWs: 58-93%, physicians: 55-98%).

For peer review only

For peer review only - http://bmjopen.bmj.com/site/about/guidelines.xhtml

Conclusions: In settings where serum bilirubin cannot be measured, neonates with any jaundice on the distal extremities should be referred to a hospital for evaluation and management. Where delays in serum bilirubin measurement and appropriate treatment are anticipated following referral, the higher-sensitivity sign, any jaundice on the distal extremities or deep jaundice of the trunk or head, may be preferred.

Article Summary

Strengths and limitations of this study'

- This study was designed to address the evidence gap from low- to middle-income countries on the reliability of clinical signs to screen newborn infants for jaundice in the first three weeks after delivery to determine the need for facility-based care, and is the largest evaluation reported to date, to our knowledge, to compare clinical assessments for jaundice in newborn infants by primary health care workers and physicians.
- The findings have the potential to be generalised to large populations in low- and middle-income countries and to influence World Health Organisation guidelines.
- We did not objectively assess the depth of jaundice, and 22% of infants with some degree of jaundice did not have laboratory work done.
- Due to the characteristics of the larger study within which this assessment was embedded, there was likely participant selection bias towards infants who were considered unwell. Thus, sensitivity and specificity may be different in other populations, in particular the general neonatal population not restricted to those considered unwell.

For peer review only

- This study did not include sufficient numbers of preterm infants to allow for independent assessment of the utility of jaundice detection in this subgroup of infants who are especially vulnerable to morbidity and mortality from hyperbilirubinemia

For peer review only

Introduction

Jaundice is among the most common neonatal problems, particularly in the first week.[1] Delayed detection and therapy place newborn infants, especially those born preterm, at risk for bilirubin encephalopathy and kernicterus, leading to neurodevelopmental impairment, disability and potentially death.[2-5] Globally in 2010 an estimated 481,000 newborn infants were at risk of extreme hyperbilirubinemia, and of these, 24% were at risk of neonatal death, 13% for kernicterus and 11% for stillbirth due to severe hemolytic disease manifest in utero as progressive anemia and hypoalbuminemia, leading to hydrops fetalis.[6]

Prevention of morbidity and mortality due to hyperbilirubinemia in low- and middle-income countries could markedly improve if primary healthcare health workers (PHWs) were able to accurately identify and appropriately refer newborn infants with clinically significant jaundice. The Integrated Management of Childhood Illness (IMCI) strategy to identify young infants with severe illness needing urgent referral-level care was revised in 2008 based on the results of the Young Infant Clinical Signs Study (YICSS),[1] and was further validated in a community-based setting in Bangladesh.[7] In 2014, IMCI included jaundice of palms and soles as a need for urgent referral.[8] Approximately three-fourths of countries have included jaundice of palms and soles in their use of IMCI, based largely on expert opinion.[9] Jaundice on the day of childbirth is always a pathological sign that requires referral to hospital for evaluation and management. Also, jaundice persisting or first presenting more than three weeks after delivery is generally pathological, and these infants also need referral for evaluation. A major gap in IMCI guidelines is the need to validate clinical signs of jaundice in newborn infants aged 1-20 days which signal urgent referral for hospital-level care.

For peer review only - http://bmjopen.bmj.com/site/about/guidelines.xhtml

Several studies have examined the correlation between identification of jaundice in newborn infants by experienced physicians and serum bilirubin levels.[10-16] The extent of peripheral spread of jaundice has been advocated as a useful clinical sign for identifying infants with high levels of serum bilirubin needing intervention.[10-12,14-17] However, others have questioned the ability of healthcare providers, including physicians and nurses, to diagnose clinical jaundice[18,19] and data are lacking on the ability of PHWs in low- and middle-income country (LMIC)[20] settings to identify newborn infants with clinically significant jaundice requiring referral for care. Point-of-care devices increasingly show promise as an alternative approach for identifying hyperbilirubinemia, but are not widely available.[21-23]

In this multicentre study nested in the YICSS,[1] we determined the sensitivity and specificity of a range of definitions of clinical jaundice, according to distribution and severity of skin staining as assessed by PHWs and by physicians, and the agreement between their clinical assessments, for predicting the presence of serum hyperbilirubinemia in neonates in the first three weeks.

Methods

Study locations and patient selection

Each of the six study locations over three continents (Table 1) collected data for a consecutive 12-month period during 2003 to 2005, as described previously.[24-27] Young infants brought to the hospital for any type of acute illness were included in the main study if they were <60 days of age and their parent or guardian provided informed consent, as described previously; the participant group for the current study was a sample of convenience based on enrolments in the

For peer review only

main study.[1] Recruitment was stratified into two age categories – 0-6 and 7-59 days – to ensure adequate sampling of neonates in the first week, when data are particularly lacking.[28] Infants presenting for care could only be enrolled once and, to ensure follow-up, had to reside in the defined study area. We excluded young infants who: 1) needed immediate cardiopulmonary resuscitation upon presentation to the hospital, 2) had an obvious lethal congenital abnormality, 3) had been hospitalised in the previous two weeks (except for delivery), or 4) were referred from another health facility.

Table 1. Methods of bilirubin detection and quality assurance by study location, Young Infants Clinical Signs Study Group.

Study location	Method for bilirubin measurement	Quality control measures
1) Dhaka Shishu Hospital, Dhaka, Bangladesh	Dimethylysulfoxide (Chronolab AG, Zug, Switzerland) colorimetric method using a Humalyzer - 2000 (Human Gesellschatt for Biochemica and Diagnostic mbH, Germany)	Quality control was done daily using calibration and quality control serum from the manufacturer
2) All India Institute of Medical Sciences and Safdarjung Hospital, Delhi, India;<br>3) Postgraduate Institute for Medical Education and Research and General Hospital, Sector 16, Chandigarh, India	Spectrophotometer (BIL-100, Cosmo Medical Co., Ltd., Seoul, Korea) with built-in auto-calibration	Bilirubin standards were used periodically (every 4-5 months) to cross-check the calibration
4) King Edward VIII Hospital, Durban, South Africa	Colorimetric method using a Roche Modular P-800 spectrophotometer (Roche Modular 800 (Roche Diagnostics, Basel, Switzerland)	The machine was manually calibrated whenever a new kit was inserted or when the machine sent out an automated request for calibration, approximately once weekly
5) Komfo Anokye Teaching Hospital, Kumasi, Ghana	Automated colorimetric method using a spectrophotometric autoanalyser (Atac-8000, Elan Diagnostics, Smithfield, RI,	The machine was calibrated daily with the manufacturer's controls

For peer review only - http://bmjopen.bmj.com/site/about/guidelines.xhtml

	USA)	
6) Hospital del Niño and Hospital Materno-Infantil, La Paz, Bolivia	Diazo method (Synchron CX5 Beckman Coulter, Inc, Fullerton, CA, USA)	Quality control was run once daily with a known sample provided by the manufacturer

Clinical assessment of jaundice

Enrolled subjects were referred to a trained PHW (i.e. a frontline professional health worker, typically a nurse) for initial evaluation. Study coordinators worked with site principal investigators to standardise their clinical assessments within and across sites. Site principal investigators in turn trained, supervised, and attempted to standardise PHWs in their assessments of jaundice. PHWs assessed infants unclothed under well-lit conditions for presence of jaundice in three body regions: 1) head, including face, gums or sclera, 2) trunk, including chest or abdomen, and 3) distal extremities, including palms or soles. If jaundice was present at any of the sites within a body region, the degree of jaundice was scored as mild or deep, taking the deepest level of staining for the sites within the body region. At Durban, the degree of jaundice was scored as mild, moderate or severe, but for analysis, moderate and severe degrees of jaundice were combined to form the “deep” category. Because sites did not start enrolling at the same time, a change in study protocol part way through the study meant that in Kumasi, which was the first site to start and complete enrollment, limited data were available from the face and chest/abdomen in comparable form to the other study sites. Data from the La Paz study location were restricted to the first half of data collection, since the distribution of serum bilirubin levels in the second half of the study suggested the introduction of a systematic error, which could not be identified and addressed, despite quality control measures; thus, data of suspect quality were excluded.

For peer review only

After the initial PHW clinical assessment, the infant was referred to a study physician for immediate evaluation and management. The physician took a complete history and performed a physical examination, blinded to the PHW's findings and to laboratory data. Signs of jaundice were assessed by physicians using the same proforma as the PHWs.

Laboratory evaluation of serum bilirubin

About 30-60 minutes after the study physician's evaluation, a blood sample was drawn from infants found to have any visible jaundice by the study physician and was tested for total serum bilirubin in the hospital laboratories (Table 1).

Patient management

Study physicians decided on the need for admission based on their clinical judgment and the serum bilirubin level. Admitted infants were managed according to the usual practices of the study hospitals.[29]

Data management and analysis

Case record forms were checked for completion and double entered into an EpiData database (version 2.1, EpiData Association, Odense, Denmark) at each of the study locations. Data files were sent to the data coordination center in Melbourne, Australia, where further data cleaning and consistency checks were performed and the quality of data submitted from the individual study locations was monitored. All cases for which there was a physician assessment were included.

For peer review only

Description of jaundice by physicians

Box-and-whisker plots were used to display the association between increasing depth and peripheral extension of jaundice (from face to trunk to distal extremities), as assessed by physicians, and serum bilirubin level. Four categories of staining were created for display: A = No jaundice, including the head; B = Mild jaundice of the head or trunk, but none of the distal extremities; C = Deep jaundice of the head or trunk, but none of the distal extremities (these data were not available for Kumasi); and D = Any jaundice of the distal extremities.

Sensitivity and specificity of jaundice definitions

A series of definitions was created based on the body site and depth of jaundice, including: (i) deep jaundice of the head, (ii) deep jaundice of the trunk, (iii) any jaundice of the distal extremities (palms, soles), (iv) any jaundice of the distal extremities OR deep jaundice of the trunk, and (v) any jaundice of the distal extremities OR deep jaundice of any site, including trunk or head. The sensitivity and specificity of classification according to these definitions of jaundice, by the PHWs and by the study physicians, were determined using thresholds for serum bilirubin as the gold standard measure as follows: (i) >260 μmol/L (>15 mg/dL), which approximates need for phototherapy according to WHO guidelines, and (ii) >340 μmol /L (>20 mg/dL), which approximates need for emergency intervention in at-risk and preterm babies.[28] All available data from PHW and physician assessments were used. Cases were classified as positive on each of the definitions assessed if any of the signs included in the definition were recorded; otherwise they were classified as negative.

Agreement between physicians and primary health care workers

For peer review only - http://bmjopen.bmj.com/site/about/guidelines.xhtml

Agreement between PHWs and physicians for identification of jaundice at each of the body regions and at each study location, for mild or severe degree of staining, was assessed by tabulating the frequency of positive classifications by each rater and using the kappa statistic. Patients aged 1-20 days were included regardless of whether they had any sign of jaundice on examination by physicians or a serum bilirubin measure. Selection of this age range was predetermined to address the gap in IMCI guidelines on clinical signs of jaundice that signal urgent referral for hospital-level care in infants aged 1-20 days.

All data analysis was performed using Stata software, Release 10 (StataCorp, College Station, TX, USA).

Data sharing

The study protocol, deidentified participant data and statistical code will be made available upon publication of the manuscript.

Ethics

The study protocol was approved by each of the sites' institutional review boards (IRB) or ethical committees. The Kumasi (#H-23160) and Durban (#H-22434) protocols were also approved by the Boston University Medical Center IRB. The Dhaka and La Paz protocols were reviewed by the Johns Hopkins University IRB and determined to be exempt from review because the protocol involved minimal risk to participants. The Delhi and Chandigarh protocols were approved by the WHO Secretariat Committee on Research Involving Human Subjects (#RPC 039/040). Informed consent was obtained for all participants.

For peer review only - http://bmjopen.bmj.com/site/about/guidelines.xhtml

Funding

The United States Agency for International Development (USAID) provided funding for this study to the Applied Research on Child Health and Child and Family Applied Research projects at Boston University, Boston, by means of the USAID cooperative agreements (HRN-A-00-90010-00 and GHS-A-00-00020-00). The Bill and Melinda Gates Foundation provided funding for Saving Newborn Lives (SNL).

Patient and public involvement

Families of study subjects were not involved in study design, recruitment, or conduct or in the dissemination of study results.

Results

Prevalence of jaundice and hyperbilirubimia

Among the 5250 young infants 1-59 days of age who were enrolled in the parent trial at the six study sites,[1] 2642 (50%) were 1-20 days of age; 1018 (38% of 2642) were young neonates < 1 week of age (days 1-6) and 1442 infants (62% of 2642) were 7-20 days of age. Dhaka had a majority (60%) of infants 1-6 days of age while Durban had a preponderance (85%) of infants 7-20 days of age (Table 2). Among infants 1-20 days of age, 8% (n=196) were preterm, ranging from 3% in La Paz to 11% in Delhi. Table S1 provides additional information on the primary diagnoses of the 2642 infants 1-20 days of age.

For peer review only - http://bmjopen.bmj.com/site/about/guidelines.xhtml

Physicians assessed that 1337 of 5250 infants (25.5%) had any sign of jaundice, 1241 of whom were aged 1-20 days (47% of 2642 babies in that age range, 93% of all young infants with jaundice) (Table 2). A total of 962 (78%) had a serum bilirubin measurement recorded, and among infants whose bilirubin level was measured, 278 (29%) and 95 (10%) had hyperbilirubinemia >260 µmol /L (>15 mg/dL) and >340 µmol /L (>20 mg/dL), respectively. The prevalence of hyperbilirubinemia varied substantially between study locations (>260 µmol /L: 10-58%, >340 µmol /L: 2-22%). Among 2608 infants aged 21-59 days (not included in this validation study), 96 (3.7%) had any sign of jaundice.

For peer review only

Table 2. Study profile: Infants aged 1-20 days assessed for jaundice and, among eligible neonates, numbers with serum bilirubin measured and with hyperbilirubinemia, by study location and overall.

	Study location						Overall
	Dhaka	Delhi	Chandigarh	Durban	Kumasi	La Paz	
Number assessed for jaundice*	448	330	700	414	457	293	2642
Preterm birth	42	36	36	42	30	10	196
% of infants	9%	11%	5%	10%	7%	3%	7%
Age group strata							
1-6 days	268	94	347	61	148	100	1,018
% of infants	60%	28%	50%	15%	32%	34%	39%
7-20 days	180	236	353	353	309	193	1,624
% of infants	40%	72%	50%	85%	68%	66%	61%
Any jaundice observed by study physicians	198	148	398	160	147	190	1241
% of assessed	44%	45%	57%	39%	32%	65%	47%
Serum bilirubin obtained	81	119	379	132	71	180	962
% of those with jaundice	41%	80%	95%	91%	48%	95%	78%
Serum bilirubin >260 µmol /l (15 mg/dL)	36	62	39	19	18	104	278
% of those with bilirubin measured	44%	52%	10%	14%	25%	58%	29%
Serum bilirubin >340 µmol /l (20 mg/dL)	14	26	9	2	8	36	95
% of those with bilirubin measured	17%	22%	2%	2%	11%	20%	10%

*By both primary healthcare workers and physicians

For peer review only - http://bmjopen.bmj.com/site/about/guidelines.xhtml

Prevalence of jaundice by depth of staining

Distributions of depth of staining by body region, according to physicians, were generally similar across locations except that the Indian physicians (especially in Delhi) recorded more deep jaundice among those found to have any jaundice (Table 3). In general, across study locations, there were decreasing proportions of subjects with deep jaundice when considering the head (22-81%) compared to the trunk (8-51%) and the distal extremities (palms/soles) (0-5%).

Table 3. Clinical signs of jaundice by body region and depth of staining among neonates aged 1-20 days with any jaundice observed by physicians, by study location.

	Infants (%) with jaundice					
	Study location					
Body region	Dhaka	Delhi	Chandigarh	Durban	Kumasi*	La Paz
Head:						
Face, gums or sclerae (*n*)	(81)	(119)	(379)	(132)	(*)	(180)
Mild	64%	19%	46%	78%		64%
Deep	36%	81%	54%	22%		36%
Trunk:						
Chest or abdomen (*n*)	(81)	(119)	(379)	(115)	(*)	(180)
None	26%	8%	24%	17%		12%
Mild	63%	40%	61%	68%		80%
Deep	11%	51%	15%	15%		8%
Distal extremities:						
Palms or soles (*n*)	(81)	(118)	(379)	(132)	(70)	(179)
None	64%	64%	93%	88%	66%	93%
Mild	35%	31%	6%	9%	34%	4%
Deep	1%	5%	1%	3%	0%	3%

* At this site, assessments were not graded as "Mild" or "Deep"; for Palms/soles the "Mild" category includes all cases with any jaundice

Association of clinical signs of jaundice with hyperbilirubinemia

Descriptive analysis

For peer review only - http://bmjopen.bmj.com/site/about/guidelines.xhtml

For peer review only

At all study locations, higher serum bilirubin levels were measured as severity and distal distribution of jaundice increased (Figure 1). However, this trend was more pronounced for some study locations (Delhi, Chandigarh) than for others (Kumasi), and the absolute levels of serum bilirubin corresponding to the different clinical jaundice categories varied between locations. At Dhaka, Durban and La Paz, there was relatively little difference in serum bilirubin levels between patients with deep jaundice of the head or of the trunk but none of the distal extremities (category C) compared to patients with jaundice extending to the distal extremities (category D).

Sensitivity and specificity

For the lower threshold of serum bilirubin (>260 μmol/L or >15 mg/dL), the highest overall sensitivity across sites was found for the definition "any jaundice of the distal extremities (palms/soles) OR deep jaundice of the trunk or head" [PHWs: 58-93% (Table 4A), physicians: 55-98% (Table 4B)]. Sensitivity for the algorithm "any jaundice of the distal extremities" was lower for PHWs (42-88%, Table 4A), and especially for physicians (11-66%, Table 4B). Sensitivity was also lower for "deep jaundice of the trunk" as reported by PHWs (26-66%, Table 4A) and physicians (14-81%, Table 4B). Sensitivity of "deep jaundice of the head" was moderate for PHWs (32-90%, Table 4A) and physicians (53-97%, Table 4B). Sensitivity of physician assessments was particularly low across the various definitions for jaundice at La Paz (11-55%, Table 4B).

For peer review only - http://bmjopen.bmj.com/site/about/guidelines.xhtml

Table 4A. Sensitivity and specificity with 95% confidence intervals (in parentheses) of clinical signs of jaundice in neonates aged 1-20 days as assessed by primary healthcare workers for detecting levels of hyperbilirubinemia.

	Sensitivity						Specificity					
	Dhaka	Delhi	Chandigarh	Durban	Kumasi	La Paz	Dhaka	Delhi	Chandigarh	Durban	Kumasi	La Paz
**Serum bilirubin > 260 mmol/l (15 mg/dl)**												
	[a](n=35)	(n=61)	(n=38)	(n=18)	(n=17)	(n=103)	(n=41)	(n=54)	(n=294)	(n=103)	(n=46)	(n=78)
**Deep jaundice of the head**	51<br>(34,69)	90<br>(80,96)	32<br>(18, 49)	56<br>(31, 78)		75<br>(65, 83)	66<br>(49, 80)	48<br>(34,62)	89<br>(85, 92)	80<br>(71, 87)		49<br>(37, 60)
**Deep jaundice of the runk**	31<br>(17,49)	66<br>(52,77)	26<br>(13, 43)	47<br>(21, 73)		31<br>(22, 41)	78<br>(62, 89)	81<br>(69,91)	96<br>(93, 98)	82<br>(72, 90)		83<br>(73, 91)
**Any jaundice of the distal extremities**	66<br>(48,81)	64<br>(51,76)	55<br>(38, 71)	56<br>(31, 78)	88<br>(64, 99)	42<br>(32, 52)	66<br>(49, 80)	80<br>(66,89)	89<br>(85, 92)	56<br>(46, 66)	76<br>(61, 87)	71<br>(59, 80)
**Any jaundice of the distal extremities OR Deep jaundice of the trunk**	66<br>(48,81)	77<br>(65,87)	58<br>(41, 74)	67<br>(41, 87)		44<br>(34, 54)	66<br>(49, 80)	72<br>(58,84)	88<br>(84, 92)	49<br>(39, 59)		69<br>(58, 79)
**Any jaundice of the distal extremities OR Deep jaundice of the head or trunk**	69<br>(51,83)	93<br>(84,98)	58<br>(41, 74)	83<br>(59, 96)		80<br>(71, 87)	63<br>(47, 78)	48<br>(34,62)	84<br>(79, 88)	48<br>(38, 58)		46<br>(35, 58)
**Serum bilirubin > 340 mmol/l (20 mg/dl)**												
	[b](n=14)	(n=25)	(n=9)	(n=2)	(n=7)	(n=35)	(n=62)	(n=90)	(n=323)	(n=119)	(n=56)	(n=146)
**Deep jaundice of the head**	79<br>(49,95)	96<br>(80,100)	67<br>(30, 93)	*		94<br>(81, 99)	66<br>(53, 78)	34<br>(25,45)	88<br>(84, 92)	76<br>(67, 83)		42<br>(34, 51)
**Deep jaundice of the trunk**	64<br>(35,87)	76<br>(55, 91)	44<br>(14, 79)			46<br>(29, 63)	82<br>(70, 91)	66<br>(55,75)	94<br>(91, 97)	77<br>(68, 85)		80<br>(73, 86)
**Any jaundice of the distal extremities**	93<br>(66,100)	80<br>(59, 93)	89<br>(52, 100)		100<br>(59, 100)	57<br>(39, 74)	61<br>(48, 73)	67<br>(56,76)	86<br>(81, 89)	55<br>(45, 64)	66<br>(52, 78)	68<br>(60, 76)
**Any jaundice of the distal extremities OR Deep jaundice of the head or trunk**	93<br>(66,100)	88<br>(69, 97)	89<br>(52, 100)			60<br>(42, 76)	61<br>(48, 73)	56<br>(45,66)	85<br>(80, 89)	46<br>(37, 56)		67<br>(59, 75)
**Any jaundice of**	93	100	89			97	58	33	81	44		38

For peer review only - http://bmjopen.bmj.com/site/about/guidelines.xhtml

**the distal extremities OR Deep jaundice of the head or trunk**	(66,100)	(86,100)	(52, 100)			(85, 100)	(45, 70)	(24,44)	(76, 85)	(35, 53)		(30, 47)

*Both cases were found to have deep jaundice of the face, and one also had jaundice of palms and soles.
[a]Numbers apply to serum bilirubin > 260 mmol/l (15 mg/dl) for sites
[b]Numbers apply to serum bilirubin > 340 mmol/l (20 mg/dl) for sites

For peer review only

Table 4B. Sensitivity and specificity with 95% confidence intervals (in parentheses) of clinical signs of jaundice in neonates aged 1-20 days as assessed by physicians for detecting levels of hyperbilirubinemia.

	Sensitivity						Specificity					
	Dhaka	Delhi	Chandigarh	Durban	Kumasi	La Paz	Dhaka	Delhi	Chandigarh	Durban	Kumasi	La Paz
**Serum bilirubin > 260 mmol/l (15 mg/dl)**												
	[a](n=36)	(n=61)	(n=39)	(n=19)	(n=17)	(n=103)	(n=46)	(n=57)	(n=340)	(n=113)	(n=53)	(n=76)
**Deep jaundice of the head**	53 (35, 70)	97 (89,100)	85 (69, 94)	79 (54, 94)		55 (45, 65)	78 (64, 89)	37 (24,51)	50 (44, 55)	88 (80, 93)		89 (80, 95)
**Deep jaundice of the trunk**	19 (8, 36)	81 (69, 90)	79 (64, 91)	44 (20, 70)		14 (8, 23)	96 (85, 99)	81 (68,90)	92 (89, 95)	90 (82, 95)		100 (95,100)
**Any jaundice of the distal extremities**	56 (38, 72)	66 (52, 77)	38 (23, 55)	42 (20, 67)	59 (33, 82)	11 (5, 18)	80 (66, 91)	95 (85,99)	97 (94, 98)	93 (87, 97)	74 (60, 85)	99 (93,100)
**Any jaundice of the distal extremities OR Deep jaundice of the trunk**	56 (38, 72)	90 (80, 96)	79 (64, 91)	47 (24, 71)		15 (9, 24)	80 (66, 91)	79 (66,89)	91 (88, 94)	87 (79, 92)		99 (93,100)
**Any jaundice of the distal extremities OR Deep jaundice of the head or trunk**	64 (46, 79)	98 (91,100)	85 (69, 94)	79 (54, 94)		55 (45, 65)	74 (59, 86)	37 (24,51)	49 (44, 55)	83 (75, 90)		88 (79, 94)
**Serum bilirubin > 340 mmol/l (20 mg/dl)**												
	[b](n=14)	(n=26)	(n=9)	(n=2)	(n=7)	(n=36)	(n=68)	(n=92)	(n=370)	(n=130)	(n=63)	(n=143)
**Deep jaundice of the head**	79 (49, 95)	100 (87,100)	100 (66, 100)	*		81 (64, 92)	74 (61, 83)	25 (16,35)	47 (42, 53)	79 (71, 86)		75 (67, 82)
**Deep jaundice of the trunk**	50 (23, 77)	92 (75, 99)	100 (66, 100)			28 (14, 45)	97 (90, 100)	60 (50,70)	87 (83, 90)	85 (77, 91)		97 (92, 99)
**Any jaundice of the distal extremities**	86 (57, 98)	85 (65, 96)	78 (40, 97)		71 (29, 96)	17 (6, 33)	75 (63, 85)	77 (67,85)	95 (92, 97)	88 (82, 93)	70 (57, 81)	96 (91, 98)
**Any jaundice of the distal extremities OR Deep jaundice of the head or trunk**	86 (57, 98)	100 (87,100)	100 (66, 100)			28 (14, 45)	75 (63, 85)	55 (44,65)	86 (82, 90)	82 (75, 88)		95 (90, 98)
**Any jaundice of**	93	100	100			81	68	24	47	75		74

For peer review only - http://bmjopen.bmj.com/site/about/guidelines.xhtml

**the distal extremities OR Deep jaundice of the head or trunk**	(66,100)	(87,100)	(66, 100)	(64, 92)	(55, 78)	(15,34)	(42, 52)	(67, 83)	(66, 81)

*Both cases were found to have deep jaundice of the face, and one also had jaundice of palms and soles.
[a]Numbers apply to serum bilirubin > 260 mmol/l (15 mg/dl) for sites
[b]Numbers apply to serum bilirubin > 340 mmol/l (20 mg/dl) for sites

For peer review only

For peer review only - http://bmjopen.bmj.com/site/about/guidelines.xhtml

Specificity of PHW assessments (Table 4A) was highest for “deep jaundice of the trunk” (78-96%) and ranged from 48-89% across all other jaundice algorithms at all study locations. Specificity varied across sites for the algorithm “any jaundice of the distal extremities or deep jaundice of the trunk or head” (46-84%). Specificity of assessments by physicians (Table 4B) was relatively high across all algorithms at all sites (74-100%) except at the Indian sites for “deep jaundice of the head” and “any jaundice of the distal extremities OR deep jaundice of the trunk or head” (37-50%).

For the higher threshold of hyperbilirubinemia (>340 μmol/L or >20 mg/dL), sensitivity was higher and specificity was similar to the findings at the lower serum bilirubin threshold for PHWs (Table 4A) and physicians (Table 4B). High sensitivity was found for “any jaundice of the distal extremities OR deep jaundice of the trunk or head” for PHWs (89-100%, Table 4A) and physicians (81-100%, Table 4B). Specificity for this algorithm was more variable for both PHWs (33-81%, Table 4A) and physicians (24-75%, Table 4B). A reasonable balance of sensitivity (71-100%) and specificity (55-95%) across both PHW and physician assessments was observed, excluding La Paz, for the definition “any jaundice of distal extremities.”

Agreement between primary healthcare workers and physicians in assessment of jaundice

Agreement between PHWs and physicians was variable for specific definitions of jaundice and across sites (Table 5). Agreement between PHWs and physicians in identifying neonates with any signs of jaundice on the head was generally good but agreement for depth of jaundice and for jaundice of the extremities was variable. Overall agreement was highest for Delhi (0.68-0.82 across jaundice algorithms).

For peer review only - http://bmjopen.bmj.com/site/about/guidelines.xhtml

Table 5. Agreement between primary healthcare workers and physicians in detecting clinical signs of jaundice in neonates aged 1-20 days, by study site.

	Study location					
	Dhaka	Delhi	Chandigarh	Durban	Kumasi	La Paz
Deep jaundice of the head	488<br>0.14 0.12<br>0.77	334<br>0.27 0.31<br>0.82	703<br>0.06 0.3<br>0.23	414<br>0.09 0.07<br>0.5		293<br>0.43 0.24<br>0.42
Deep jaundice of the trunk (chest or abdomen)	488<br>0.08 0.02<br>0.43	334<br>0.15 0.19<br>0.73	703<br>0.03 0.08<br>0.35	323<br>0.08 0.05<br>0.61		293<br>0.16 0.05<br>0.44
Any jaundice of the distal extremities (palms or soles)	488<br>0.15 0.10<br>0.61	333<br>0.16 0.13<br>0.68	703<br>0.08 0.04<br>0.41	414<br>0.17 0.04<br>0.29	456<br>0.16 0.12<br>0.49	292<br>0.24 0.04<br>0.21

Data for each definition of clinical jaundice are displayed as follows: (row 1) number of infants evaluated by both observers; (row 2) proportion of infants judged positive by primary healthcare workers and by physicians, respectively; (row 3) kappa statistic for measuring agreement beyond chance.

Discussion

The study is the largest evaluation reported to date on the validity of clinical assessments for jaundice by PHWs and physicians in identifying neonates with hyperbilirubinemia at levels indicating need for referral-level care. We observed that a finding of "any jaundice of the distal extremities" (palms or soles) by PHWs or physicians had sensitivity and specificity generally exceeding about 70% across locations for detecting neonates aged 1-20 days with bilirubin levels >340 µmol /L (20 mg/dL) – a level that requires acute intervention in a health facility, including evaluation for plasma exchange transfusion in at-risk and preterm babies. Given that this level of jaundice places infants at risk of severe morbidity, we also note that the broader algorithm "any jaundice of the distal extremities OR deep jaundice of the trunk or head" had high sensitivity for both PHWs (89-100%) and physicians (81-100%), although with variable specificity across study sites (33-81% for PHWs, 24-75% for physicians). Use of this latter algorithm could result

For peer review only

For peer review only - http://bmjopen.bmj.com/site/about/guidelines.xhtml

in the referral of more at-risk infants (based on high sensitivity), but also, depending on specificity at the site, more infants without significant hyperbilirubinemia. This latter algorithm may be preferable in settings in which prompt referral and access to timely measurement and management of hyperbilirubinemia are lacking or anticipated to be delayed, such as in many LMIC peripheral healthcare settings with minimal equipment and skilled providers where use of the higher sensitivity algorithm may minimise missing patients who can benefit from initiation of treatment while referral is managed. Clinical signs were also discriminatory, but less so, for identifying infants who had moderately elevated bilirubin (>260 μmol/L). For this lower threshold level indicating need for phototherapy, the algorithm "any jaundice of the distal extremities OR deep jaundice of the trunk or head" also had the highest overall sensitivity across sites (58-93% for PHWs and 55-98% for physicians). Thus, in settings where it is not possible to measure serum bilirubin, such as in many peripheral health facilities in LMICs which use IMCI, it is recommended to refer neonates aged 1-20 days with yellow palms and soles or deep jaundice on the trunk or head to a hospital for further evaluation and management.

We found very few cases in which jaundice appeared on the first day after delivery or lasted beyond three weeks of age; all these babies should be referred to a health facility for further assessment and management, including direct and total bilirubin measurement. A small percentage of infants aged 21-59 days had jaundice (n=96, 3.7%); jaundice presenting in this age group is presumed pathological and all such infants should also be referred for further clinical and laboratory workup.

For peer review only - http://bmjopen.bmj.com/site/about/guidelines.xhtml

Several previous studies have assessed the value of clinical signs for predicting high levels of serum bilirubin in neonates.[10-19] In many LMICs where births commonly occur either at home or at peripheral health clinics, laboratory measurement of serum bilirubin may not be easily available and clinical signs have to be used as a basis for referral. Similar to some previous studies in LMICs, we found that serum bilirubin levels were generally higher in neonates with jaundice of more caudal parts of the body.[12-16] Jaundice extending most distally to the palms and soles was most discriminatory in identifying neonates with very high levels of bilirubin in need of urgent evaluation for treatment, and thus, has clinical relevance. However, jaundice that deeply involved the trunk or head was also useful clinically for identification of patients with hyperbilirubinemia requiring treatment and recognition of this more sensitive sign, as part of the algorithm "any jaundice of the distal extremities or deep jaundice of the trunk or head" may also be important in low resource settings with fewer diagnostic and treatment capabilities.

In general, PHWs had higher sensitivity than physicians in identifying neonates with hyperbilirubinemia based on identification of clinical signs of jaundice. Overall, the sensitivity of any jaundice on palms and soles in detecting infants with high bilirubin levels >340 µmol /L was higher for PHWs than for physicians, but the specificity was about 10 percentage points lower in most locations. For identifying infants with bilirubin levels >260 µmol /L, specificity was slightly higher for PHWs, while sensitivity was similar for both cadres. These results emphasise that once neonates with suspected hyperbilirubinemia based on PHW clinical asserssment reach a health facility, further physician clinical assessment and laboratory evaluations must be performed whenever possible to determine need for treatment. We observed considerable variation in the performance of clinical signs of jaundice between study locations, despite

For peer review only - http://bmjopen.bmj.com/site/about/guidelines.xhtml

attempting to standardise PHW training and physician supervision. Overall, clinical signs had lower sensitivity in La Paz and lower specificity in Dhaka and Durban, and agreement for the presence of jaundice on the palms and soles was lowest in Durban and La Paz compared to the other locations. These findings highlight the importance of local evaluation and adaptations of algorithms.

Variable skin pigmentation is an important potential confounding factor in the clinical detection of jaundice.[13] In Kumasi, where skin pigmentation was darkest among the study locations, study investigators were not confident that they could identify jaundice on the face, chest or thighs; as a result, their assessments of jaundice were limited to the sclerae, gums, palms and soles. Nevertheless, PHWs in Kumasi were able to identify neonates needing referral for hyperbilirubinemia based on recognition of jaundice on the palms and soles, with good sensitivity and reasonable specificity.

This study had several strengths including its size (n=1242) and multiple sites, jaundice assessment at several locations on each infant, and systematic evaluation of PHW assessments of jaundice. The findings have the potential to be generalised to large populations in LMICs.

This study had several limitations. We did not attempt to objectively assess the depth of jaundice, for example by the use of color scales for comparison. This reflects the reality in many LMIC settings where such scales or visual cue cards are not available, however, their use could be a valuable addition in future research. Also, due to inconsistencies in obtaining blood samples for bilirubin measurement, 22% of infants with some degree of jaundice did not have laboratory

For peer review only - http://bmjopen.bmj.com/site/about/guidelines.xhtml

work done. In addition, the decision to draw blood for bilirubin detection was based on the study physicians' diagnoses and not the PHWs' assessments. Given that PHWs had higher sensitivity than physicians in identifying neonates with hyperbilirubinemia based on identification of clinical signs of jaundice, some infants with jaundice identified by PHWs did not have blood samples obtained. Since the study design focused on sick young infants presenting for care, asymptomatic infants with jaundice may not have been brought for assessment, and thus, the sensitivity and specificity of clinical assessments may vary at population level and warrant further investigation. However, infants presenting for routine health care (e.g., well baby checks) may also be at risk for complications of jaundice and therefore should be screened. We did not identify the characteristics of the newborns that are prone to screening errors, which we recooomend for future research to further improve algorithm performance. This study did not include sufficient numbers of preterm infants to allow for independent assessment of the utility of jaundice detection in this subgroup of infants, who are especially vulnerable to morbidity and mortality from hyperbilirubinemia. It is challenging to accurately identify gestational age in LMICs where ultrasound assessment may not be available. Therefore, a more conservative approach to referral of jaundiced infants suspected to be preterm is advisable, for example use of the algorithm "any jaundice of the distal extremities or deep jaundice of the trunk or head." Finally, there were wide variations between sites in the proportion of jaundiced infants who had a bilirubin measured (41-95%) and in the proportion of bilirubin levels that were severe (2-22% >340 μmol/l). Reasons for variation in severity of hyperbilirubinemia by site are not known, but could reflect local differences in underlying risk factors as well as access to and timeliness of care-seeking. While the potential impact of these issues on our findings are unclear, current

For peer review only - http://bmjopen.bmj.com/site/about/guidelines.xhtml

estimates of sensitivity and specificity of visual appraisal for identifying infants with various thresholds of hyperbilirubinemia should be interpreted with some caution.

Conclusion

Further standardisation of training to ensure greater consistency among PHWs' assessments, as well as local research and monitoring in settings where IMCI guidelines are implemented would be useful to further refine the assessment and referral criteria for jaundice. The current IMCI algorithm focuses on jaundice of palms and soles as indicative of need for urgent referral. However, the more broadly defined algorithm "any jaundice of the distal extremities deep jaundice of the trunk or head" may identify more at-risk neonates – although at a potential cost of referral of greater numbers of infants not needing acute intervention (e.g., phototherapy, exchange transfusion) – and may be preferred in settings where delay in receiving care is anticipated. Local evidence-based adaptations of these algorithms are needed, particularly regarding consideration of deep staining of the trunk or head and the balance between increased sensitivity, enabling the detection of greater numbers of infants needing further assessment, and increased specificity and targeting of scare diagnostic and treatment resources. Many hospitals in LMICs cannot determine bilirubin levels reliably.[30] In addition to building basic laboratory capacity in developing country hospitals, further refinement and evaluation of the utility of point-of-care technologies[21-23,31] to augment clinical assessment for detection of hyperbilirubinemia is warranted.

For peer review only - http://bmjopen.bmj.com/site/about/guidelines.xhtml

Acknowledgements

We thank the local communities at the study sites for their participation in the study, and also thank all members of the Young Infant Clinical Signs Study Group (YICSSG) who made this study possible.

YICSSG members include (exclusive of named co-authors):

Study sites: Bangladesh: *Clinical investigators:* A. S. M. Nawshad Uddin Ahmed, Md. Monir Hossain; *Study physicians:* Nazmun Nahar; *Nurses*: Amala Baidya, Mahmuda Parul; *Laboratory personnel*: Maksuda Islam, Tania Nasreen; *Data management*: Md. Rezaur Rahaman; **Bolivia**: *Study physicians*: Teresa Villagomez, Pablo Mattos; Manuel Pantoja Ludueña, Remedios Zumarán; *Study nurses*: Irma Quispe, Willy Tarqui, Lourdes Checa, Claudia Canqui; *Data management:* Erick Dueñas, Omar Vargas; **Ghana**: *Study physicians:*G. Plange-Rhule, Osei Akoto; *Laboratory:* M. Lartey; *Data management:* Henrietta Akpene; **India, Chandigarh:** *Clinical investigators:* Rupinder Narang; *Study physicians:* Prasad Muley, Satish Misra; *Nurses:* Tapasaya, Sanjay Rani; *Laboratory:* Pallab Ray, Tamanna Gaur; *Data management:* Vishal Kanojia, Ajay Dogra; **India, Delhi:** *Clinical investigators:* Harish Chellani, M. S. Prasad; *Study physician:* A. Satyavani; *Nurses:* Jyoti, Raji John; *Laboratory:* Arti Kapil; *Data management:* Sanjeev Negi, Narinder Singhal**; South Africa:** *Clinical investigator:* Mirian Adhikari; *Nurse:* Sister Mojaphelo; *Laboratory:* Wim Sturm; *Data management:* Precious Sikhakhane.

Study advisors: Kim Mulholland, Vinod Paul, Eric Simoes, Jelka Zupan

Data Management: Philip Greenwood, Claudine Chionh, Murdoch Children's Research Institute.

For peer review only

References

1. Young Infants Clinical Signs Study Group (YICSSG). Clinical signs predicting severe illness in young infants: a multicentre study. Lancet 2008;371:135-42.

2. Cashore WJ. The neurotoxicity of bilirubin. Clin Perinatol 1990;17:437-47.

3. Ip S, Chung M, Kulig J, O'Brien R, Sege R, Glicken S, et al. American Academy of Pediatric Technical Report. An evidence-based review of important issues concerning neonatal hyperbilirubinemia. Pediatrics 2004;114:e130-153.

3. Totapally BR, Torbati D. Neonatal jaundice. Int Pediatr 2005;20:47-54.

5. Maulik PK, Darmstadt GL. Childhood disability in low and middle income countries: overview of screening, prevention, services, legislation and epidemiology. Pediatrics 2007;120(Suppl 1);S1-S55.

6. Bhutani VK, Zipursky A, Blencowe H, Khanna R, Sgro M, Ebbesen F, et al. Neonatal hyperbilirubinemia and Rhesus disease of the newborn: incidence and impairment estimates for 2010 at regional and global levels. Pediatr Res 2013;74:86-100.

7. Darmstadt GL, Baqui AH, Choi Y, Bari S, Rahman SM, Mannan I, et al. Validation of community health workers' assessment of neonatal illness in rural Bangladesh. Bull World Health Organ 2009;87:12-19.

8. World Health Organization WH. IMCI chart booklet. World Health Organization, Geneva, Switzerland. 2014.

9. Boschi-Pinto C, Labadie G, Dilip TR, Oliphant N, Dalglish SL, Aboubaker S, et al. Global implementation survey of Integrated Management of Childhood Illness (IMCI): 20 years on. BMJ Open. 2018;8(7):e019079.

For peer review only

10. Goswami V, Dutta AK, Singh V, Chandra J. Evaluation of simple clinical signs of illness in young infants (0-2 months) and its correlation with WHO IMCI algorithm (7 days to 2 months). Indian Pediatr 2006;43:1042-1049.

11. Hatzenbuehler L, Zaidi AK, Sundar S, Sultana S, Abbasi F, A Rizvi, Darmstadt GL. Validity of neonatal jaundice evaluation by primary health-care workers and physicians in Karachi, Pakistan. J Perinatol 2010;30(9):616-.

12. Ebbesen F. Relationship between the cepahlo-pedal progress of clinical icterus and the serum bilirubin concentration in newborn infants without blood type sensitization. Acta Obstet Gynecol Scand 1975;54:329-332.

13. Knudsen A, Brodersen R. Skin colour and bilirubinemia in neonates. Arch Dis Child 1989;64:605-9.

14. Madlon-Kay DJ. Recognition of the presence and severity of newborn jaundice by parents, nurses, physicians, and icterometer. Pediatrics 1997;100(3):E3.

15. Madlon-Kay DJ. Home health nurse clinical assessment of neonatal jaundice: comparison of three methods. Arch Pediatr Adolesc Med 2001;155:583-6.

16. Riskin A, Kuglman A, Abend-Weinger M, Green M, Hemo M, Bader D. In the eye of the beholder: how accurate is clinical estimation of jaundice in newborns? Acta Paediatr 2003;92:574-576.

17. Kramer LI. Advancement of dermal icterus in the jaundiced newborn. Amer J Dis Child 1969;118:454-458.

18. Moyer VA, Ahn C, Sneed S. Accuracy of clinical judgement in neonatal jaundice. Arch Pediatr Adolesc Med 2000;154:391-4.

For peer review only - http://bmjopen.bmj.com/site/about/guidelines.xhtml

19. Tayaba R, Gribetz D, Gribetz I, Holzman IR. Noninvasive estimation of serum bilirubin. Pediatrics 1998;102(3):E28.

20. Serajuddin U, Hamadeh N. New World Bank country classifications by income level: 2020-2021. https://blogs.worldbank.org/opendata/new-world-bank-country-classifications-income-level-2020-2021 (accessed 18 August 2021).

21. Boo NY, Chang YF, Leong YX, Tok ZY, Hooi LC, Che SC, Latif ZA. The point-of-care Bilistick method has very short turn-around-time and high accuracy at lower cutoff levels to predict laboratory-measured TSB. Pediatr Res 2019 Aug;86(2):216-220. doi: 10.1038/s41390-019-0304-0.

22. Rohsiswatmo R, Oswari H, Amandito R, Sjakti HA, Windiastuti E, Roeslani RD, Barchia I. Agreement test of transcutaneous bilirubin and bilistick with serum bilirubin in preterm infants receiving phototherapy BMC Pediatr 2018 Sep 29;18(1):315. doi: 10.1186/s12887-018-1290-9.

23. Greco C, Iskander IF, Houchi SZE, Rohsiswatmo R, Rundjan L, Ogala WN, et al. Diagnostic performance analysis of the point-of-care Bilistick system in identifying severe neonatal hyperbilirubinemia by a multi-country approach. EClinicalMedicine 2018 Jul 17;1:14-20. doi: 10.1016/j.eclinm.2018.06.003.

24. Deorari AK, Chellani H, Carlin JB, Greenwood P, Prasad MS, Satyavani A, et al. Clinicoepidemiological profile and predictors of severe illness in young infants (<60 days) reporting to a hospital in North India. Indian Pediatr 2007;44:739-748.

25. Narang A, Kumar P, Narang R, Ray P, Carlin JB, Greenwood P, et al. Clinico-epidemiological profile and validation of symptoms and signs of severe illness in young

For peer review only

For peer review only - http://bmjopen.bmj.com/site/about/guidelines.xhtml

infants (<60 days) reporting to a district hospital in North India. Indian Pediatr 2007;44:751-759.

26. Yeboah-Antwi K, Addo-Yobo E, Adu-Sarkodie Y, Carlin JB, Plange-Rhule G, Osei Akoto A, et al. Clinico-epidemiological profile and predictors of severe illness in young infants (0-59 days) in Ghana. Ann Trop Paediatr 2008;28:35-43.

27. Jeena PM, Adhikari M, Carlin JB, Qazi S, Weber MW, Hamer DH. Clinical profile and predictors of severe illness in young South African infants (<60 days). S Afr Med J. 2008;98(11):883-888.

28. World Health Organization Young Infants Study Group. Methodology for a multicenter study of serious infections in young infants in developing countries. Pediatric Infectious Diseases Journal 1999;10 (Suppl.):S8-S16.

29. World Health Organization. Pocket book of hospital care for children: Guidelines for the management of common childhood illnesses, second edition. WHO Geneva 2013, ISBN: 978 92 4 154837 3.

30. Nolan T, Angos P, Cunha AJ, Muhe L, Qazi S, Simoes EA, et al. Quality of hospital care for seriously ill children in developing countries. Lancet 2001;357(9250):106-10.

31. Lee AC, Folger LV, Rahman M, et al. A novel icterometer for hyperbilirubinemia screening in low-resource settings. Pediatrics. 2019;143(5):e20182039.

For peer review only

For peer review only - http://bmjopen.bmj.com/site/about/guidelines.xhtml

Author Contributions

GLD, DHH, JBC and MWW co-conceived the study design, planned and prepared study protocols, and provided oversight to overall implementation, data collection, data analysis, and interpretation of data. Site PIs provided input to study design, and directly coordinated and provided oversight to study implementation, data collection, data reporting, and interpretation of data at Chandigarh (PK, AN, PR), Delhi (AD), Kumasi (EAY, YAS, KYA), Durban (PMJ), Bangladesh (MAKAC, SKS), and La Paz (AB and EM). RB contributed to data analysis and interpretation of data. JBC led analysis of data across the study sites. EMF contributed to literature review and interpretation of data. GLD wrote the first draft of the manuscript. All authors reviewed and provided input to the manuscript and approved the paper for publication.

Competing interests

RB and MWW are WHO staff members. MWW contributed to study design, implementation, data analysis and manuscript writing and RB contributed to data analysis and manuscript writing. The opinions expressed in this paper do not necessarily represent the position of the WHO. The funding agencies did not influence the conduct or outcomes of the study, data analysis or interpretation, or preparation of this paper. GLD, DHH, JBC, and MWW had access to raw data. The corresponding author had full access to all data and had final responsibility for the decision to submit the paper for publication.The authors declare no other potential competing interests.

Patient consent for publication

Not required.

Provenance and peer review

Not commissioned; externally peer reviewed.

For peer review only

For peer review only - http://bmjopen.bmj.com/site/about/guidelines.xhtml

Figure legends

Figure 1. Box plots of serum bilirubin levels in neonates aged 1-20 days according to clinical signs of jaundice detected by physicians, by study location. Horizontal lines indicate the two thresholds for hyperbilirubinemia used in subsequent analysis [260 umol/L (15 mg/dL) and 340 (20 mg/dL) umol/L).

For peer review only

Figure 1. Box plots of serum bilirubin levels in neonates aged 1-20 days according to clinical signs of jaundice detected by physicians, by study location. Horizontal lines indicate the two thresholds for hyperbilirubinemia used in subsequent analysis (260 and 340 umol/L).

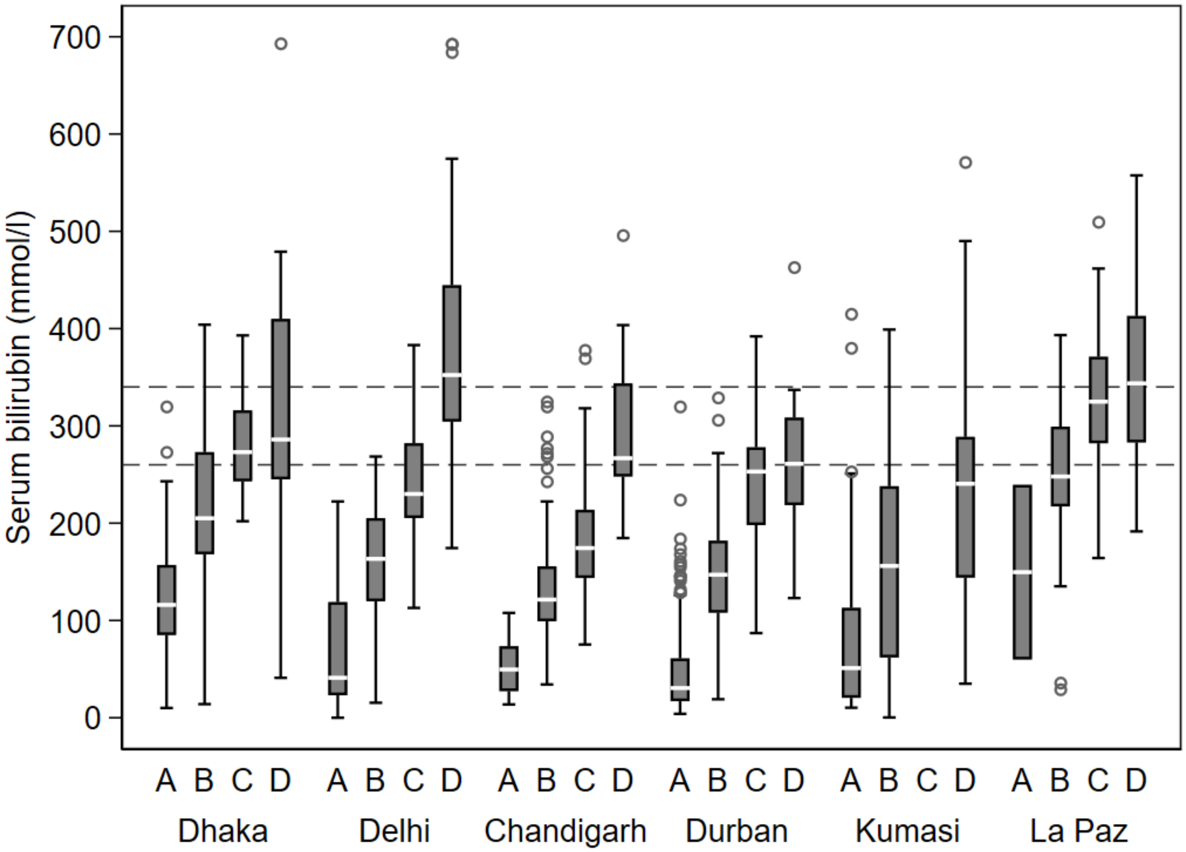

Table S1. Primary diagnoses of infants 1-20 days of age who presented to Young Infant Clinical Signs Study Group sites for evaluation

Primary diagnosis	N	%	Cumulative %
Jaundice	756	28.6	28.6
URTI / mild ARI	233	8.8	37.4
Skin infection	195	7.4	44.8
Eye infection	189	7.2	52.0
Sepsis	166	6.3	58.2
Feeding problem	94	3.6	61.8
Gastrocolic reflex	83	3.1	65.0
Birth asphyxia	65	2.5	67.4
Congenital malformation	61	2.3	69.7
Skin condition (non-infectious)	59	2.2	72.0
Birth injury	58	2.20	74.2
Colic	48	1.8	76.0
Diarrhea	45	1.70	77.7
Prematurity	41	1.6	79.2
Umbilical infection	41	1.6	80.8
Pneumonia / ALRI	37	1.4	82.2
Oral thrush	29	1.1	83.3
Cephalohematoma	25	1.0	84.2
Nasolacrimal duct block	20	0.8	85.0
High environmental temperature	17	0.6	85.6
Regurgitation	16	0.6	86.2
Umbilical problem	16	0.6	86.8
Low birth weight	14	0.5	87.4
Seizures	12	0.4	87.8
Breast abscess	12	0.4	88.3
Meningitis	9	0.3	88.6
Constipation	9	0.3	89.0
Hypoglycaemia	5	0.2	89.1

For peer review only

Laryngomalacia	5	0.2	89.3
Transient tachypnoea	3	0.1	89.4
Meconium aspiration syndrome	3	0.1	89.6
Hypothermia	3	0.1	89.7
Dysentery (bloody stools)	3	0.1	89.8
Intestinal obstruction	3	0.1	89.9
None	150	5.7	96.1
Other (≤ 2 cases)	117	4.4	---
Total	2,642	100.0	

*Note that percentages are rounded off to the nearest 0.1; they add to 100% over the entire sample

Abbreviations: ALRI, acute lower respiratory infection; ARI, acute respiratory infection; URTI, upper respiratory tract infection

For peer review only

STROBE Statement—checklist of items that should be included in reports of observational studies

	Item No	Recommendation	
**Title and abstract**	1	(*a*) Indicate the study's design with a commonly used term in the title or the abstract	Title, p. 1
		(*b*) Provide in the abstract an informative and balanced summary of what was done and what was found	Abstract, p. 3-4
**Introduction**			
Background/rationale	2	Explain the scientific background and rationale for the investigation being reported	Introduction, pp. 5-6
Objectives	3	State specific objectives, including any prespecified hypotheses	Introduction, p. 6
**Methods**			
Study design	4	Present key elements of study design early in the paper	Methods, pp. 6-9
Setting	5	Describe the setting, locations, and relevant dates, including periods of recruitment, exposure, follow-up, and data collection	Methods, pp. 6-9, Table 1
Participants	6	(*a*) *Cohort study*—Give the eligibility criteria, and the sources and methods of selection of participants. Describe methods of follow-up<br>*Case-control study*—Give the eligibility criteria, and the sources and methods of case ascertainment and control selection. Give the rationale for the choice of cases and controls<br>*Cross-sectional study*—Give the eligibility criteria, and the sources and methods of selection of participants	Methods, pp. 6-7
		(*b*) *Cohort study*—For matched studies, give matching criteria and number of exposed and unexposed<br>*Case-control study*—For matched studies, give matching criteria and the number of controls per case	N/A
Variables	7	Clearly define all outcomes, exposures, predictors, potential confounders, and effect modifiers. Give diagnostic criteria, if applicable	Methods, pp. 8-9
Data sources/ measurement	8*	For each variable of interest, give sources of data and details of methods of assessment (measurement). Describe comparability of assessment methods if there is more than one group	Methods, pp. 8-9
Bias	9	Describe any efforts to address potential sources of bias	Methods, p. 8
Study size	10	Explain how the study size was arrived at	Methods, p. 6

For peer review only - http://bmjopen.bmj.com/site/about/guidelines.xhtml
For peer review only

Quantitative variables	11	Explain how quantitative variables were handled in the analyses. If applicable, describe which groupings were chosen and why	Methods, pp. 9-10
Statistical methods	12	(*a*) Describe all statistical methods, including those used to control for confounding	Methods, pp. 9-10
		(*b*) Describe any methods used to examine subgroups and interactions	N/A
		(*c*) Explain how missing data were addressed	pp. 9-10
		(*d*) *Cohort study*—If applicable, explain how loss to follow-up was addressed<br>*Case-control study*—If applicable, explain how matching of cases and controls was addressed<br>*Cross-sectional study*—If applicable, describe analytical methods taking account of sampling strategy	N/A
		(*e*) Describe any sensitivity analyses	N/A

Continued on next page

For peer review only

For peer review only - http://bmjopen.bmj.com/site/about/guidelines.xhtml

**Results**			
Participants	13*	(a) Report numbers of individuals at each stage of study—eg numbers potentially eligible, examined for eligibility, confirmed eligible, included in the study, completing follow-up, and analysed	Results, p. 12, Tables 2-5
		(b) Give reasons for non-participation at each stage	N/A
		(c) Consider use of a flow diagram	Summarized p. 12, Table 2
Descriptive data	14*	(a) Give characteristics of study participants (eg demographic, clinical, social) and information on exposures and potential confounders	See parent study, reference 1
		(b) Indicate number of participants with missing data for each variable of interest	Results, Tables 2-5
		(c) *Cohort study*—Summarise follow-up time (eg, average and total amount)	Methods, p. 9
Outcome data	15*	*Cohort study*—Report numbers of outcome events or summary measures over time	Tables 2-5
		Case-control study—Report numbers in each exposure category, or summary measures of exposure	N/A
		Cross-sectional study—Report numbers of outcome events or summary measures	N/A
Main results	16	(*a*) Give unadjusted estimates and, if applicable, confounder-adjusted estimates and their precision (eg, 95% confidence interval). Make clear which confounders were adjusted for and why they were included	N/A
		(*b*) Report category boundaries when continuous variables were categorized	Results, pp. 13-18
		(*c*) If relevant, consider translating estimates of relative risk into absolute risk for a meaningful time period	N/A
Other analyses	17	Report other analyses done—eg analyses of subgroups and interactions, and sensitivity analyses	N/A
**Discussion**			
Key results	18	Summarise key results with reference to study objectives	Discussion, pp. 13-18
Limitations	19	Discuss limitations of the study, taking into account sources of potential bias or imprecision. Discuss both direction and magnitude of any potential bias	Discussion, p. 21-22
Interpretation	20	Give a cautious overall interpretation of results considering objectives, limitations, multiplicity of analyses, results from similar studies, and other relevant evidence	Discussion, pp. 18-23
Generalisability	21	Discuss the generalisability (external validity) of the study results	Discussion, pp. 18-23; Conclusion, pp. 22-23
**Other information**			
Funding	22	Give the source of funding and the role of the funders for the present study and, if applicable, for the original study on which the present article is based	p. 11

For peer review only

*Give information separately for cases and controls in case-control studies and, if applicable, for exposed and unexposed groups in cohort and cross-sectional studies.

Note: An Explanation and Elaboration article discusses each checklist item and gives methodological background and published examples of transparent reporting. The STROBE checklist is best used in conjunction with this article (freely available on the Web sites of PLoS Medicine at http://www.plosmedicine.org/, Annals of Internal Medicine at http://www.annals.org/, and Epidemiology at http://www.epidem.com/). Information on the STROBE Initiative is available at www.strobe-statement.org.

For peer review only

For peer review only - http://bmjopen.bmj.com/site/about/guidelines.xhtml
